# Supplementary material for: The Tharsis mantle source of depleted shergottites revealed by 90 million impact craters
Source: Nat Commun. 2021 Nov 3;12:6352. doi: 10.1038/s41467-021-26648-3 (PMC8566585; doi:10.1038/s41467-021-26648-3)
Supplement: Supplementary file 1 — Supplementary Information [file 41467_2021_26648_MOESM1_ESM.pdf]

Supplementary Information for

The Tharsis mantle source of depleted shergottites revealed by 90 million impact craters

A. Lagain*, G. K. Benedix, K. Servis, D. Baratoux, L. S. Doucet, A. Rajšic, H. A. R. Devillepoix, P. A. Bland, M. C. Towner, E. K. Sansom and K. Miljković.

* Corresponding author: anthony.lagain@gmail.com

This PDF includes:

- Supplementary Figures 1-47
- Supplementary Table 1-4
- Supplementary References

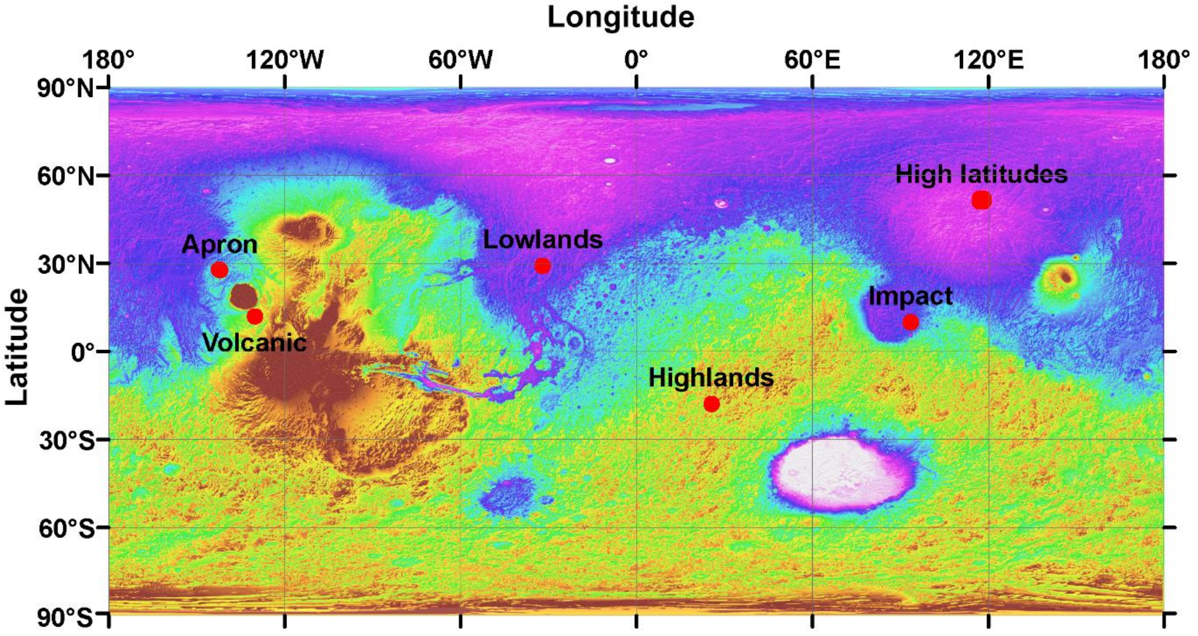

Supplementary Figure 1. Location of the CDA results evaluation. Impact craters on six different type of terrains are mapped and compared to the automatic detections. Background: topography map from the Mars Orbital Laser Altimeter (MOLA) dataset, https://planetarymaps.usgs.gov/mosaic/Mars_MGS_MOLA_DEM_mosaic_global_463m.tif.

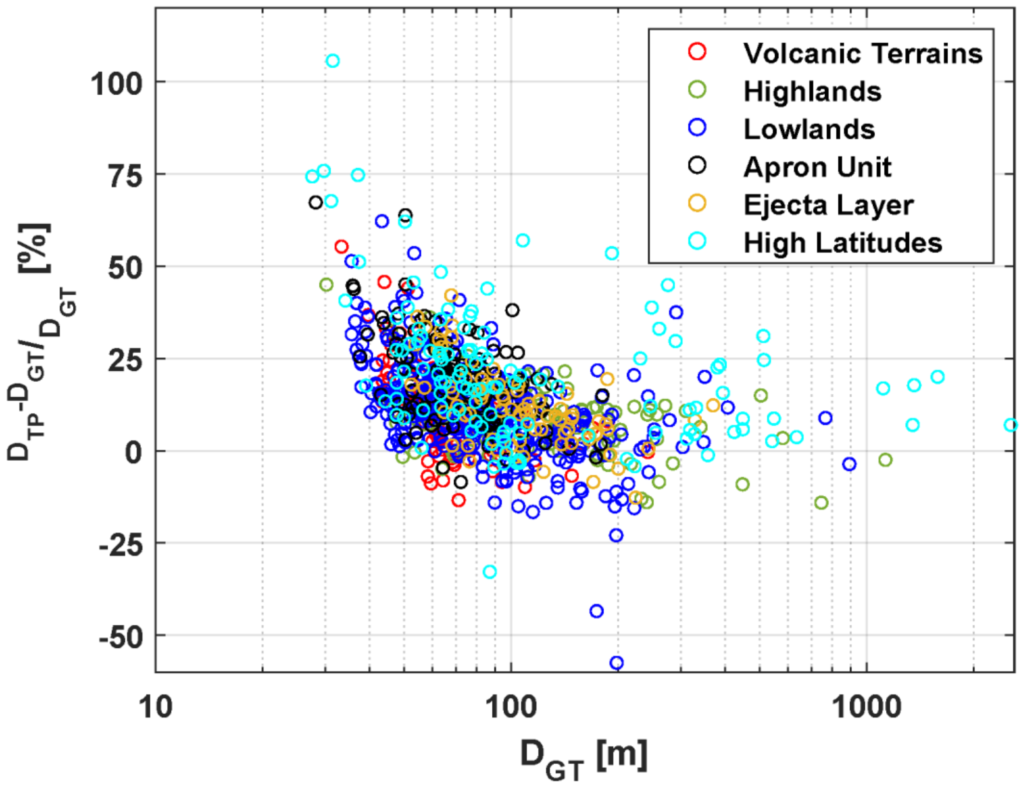

Supplementary Figure 2. Evaluation of the diameter estimation uncertainty from the CDA on different type of terrains. Diameter difference between true positive detections (TP) compared to the same crater manually mapped (Ground Truth or GT). Above 0%, the diameter measured by the CDA is overestimated, while below 0%, the CDA underestimates the crater diameter. Note that from 100m, the crater diameter estimated by the CDA is within ±25% of that manually measured. The different terrains location are shown on Supplementary Figure 1.

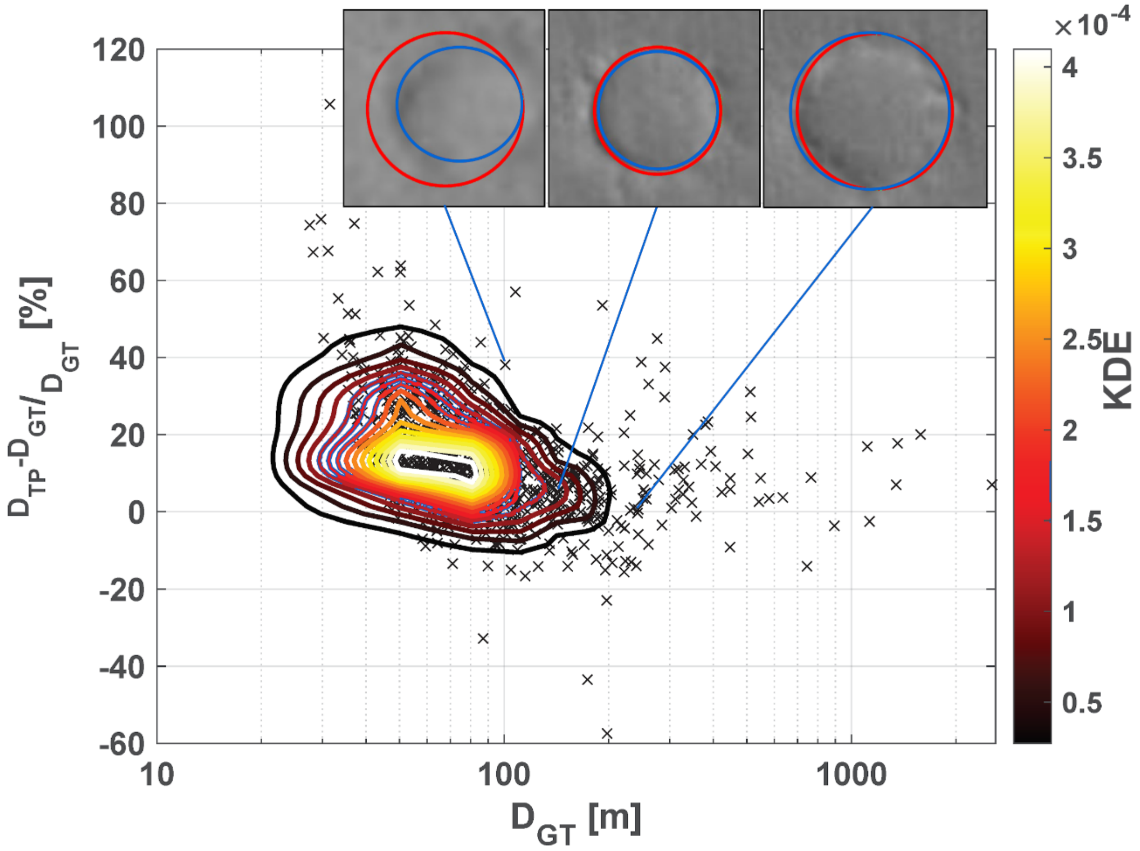

Supplementary Figure 3. Kernel density estimator (KDE) of the diameter estimation uncertainty. The diameter difference between impact craters detected by the CDA (True Positive or TP) are compared to their manually mapped counterpart (Ground Truth or GT) and the kernel density estimator shows that detections larger than 100m are estimated within 25% of uncertainties compared to the diameter measured manually. The close-ups illustrate the detections (in red), seen in CTX, compared to their manual counterpart (in blue). Readers are referred to the X-axis for the scale of each image.

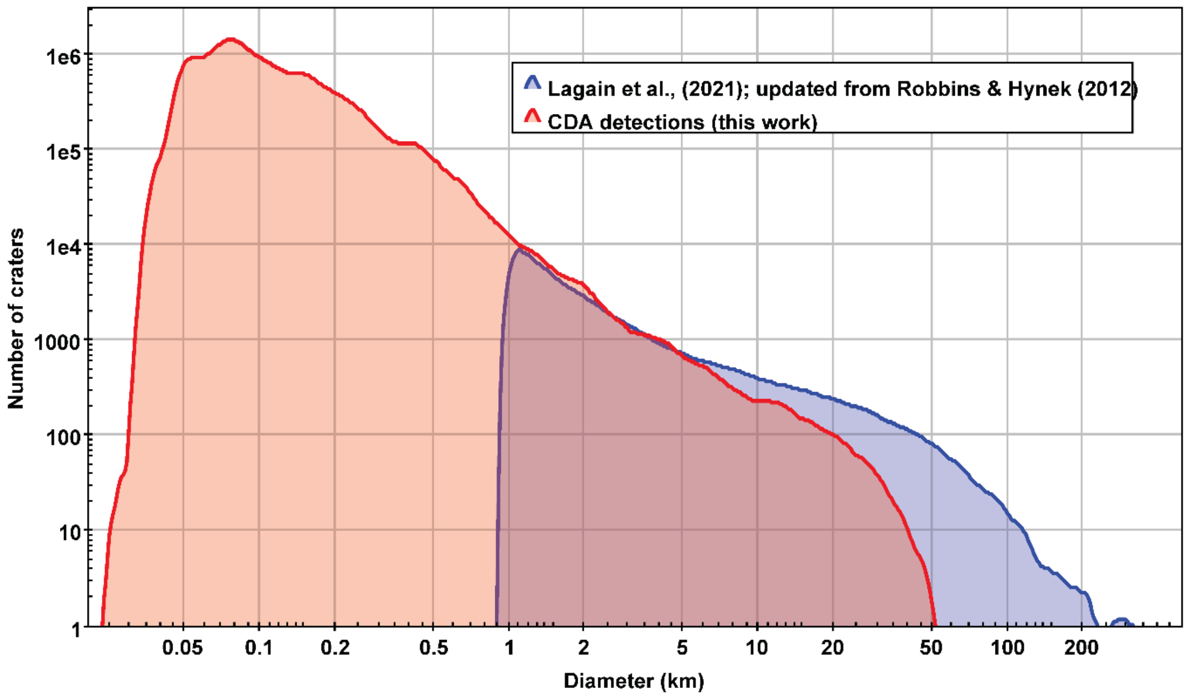

Supplementary Figure 4. Number and size of craters detected by the CDA compared to the manual database complete down to 1 km in diameter. Crater Size-Frequency Distribution of the automatic crater database presented in this work (in red) compared to the manual catalogue (in blue) from[1-2].

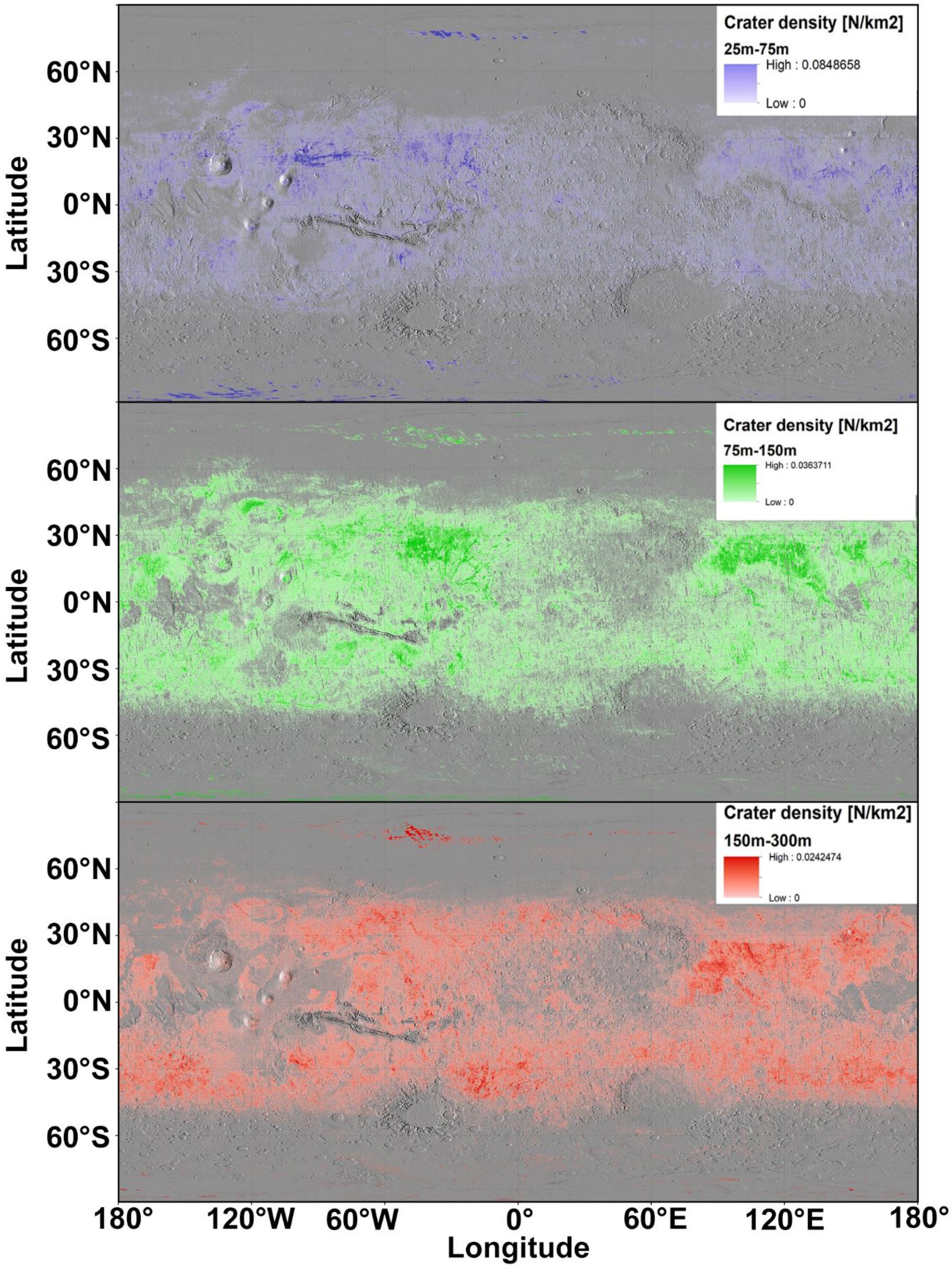

Supplementary Figure 5. Crater density maps of each band of the RGB map presented on Fig. 1. The cratering density distribution is computed for each size range, from the top to the bottom: 25 m < D < 75 m (from 0 to 0.085 craters/km^2), 75 m < D < 150 m (from 0 to 0.036 craters/km^2), 150 m < D < 300 m (from 0 to 0.024 craters/km^2).

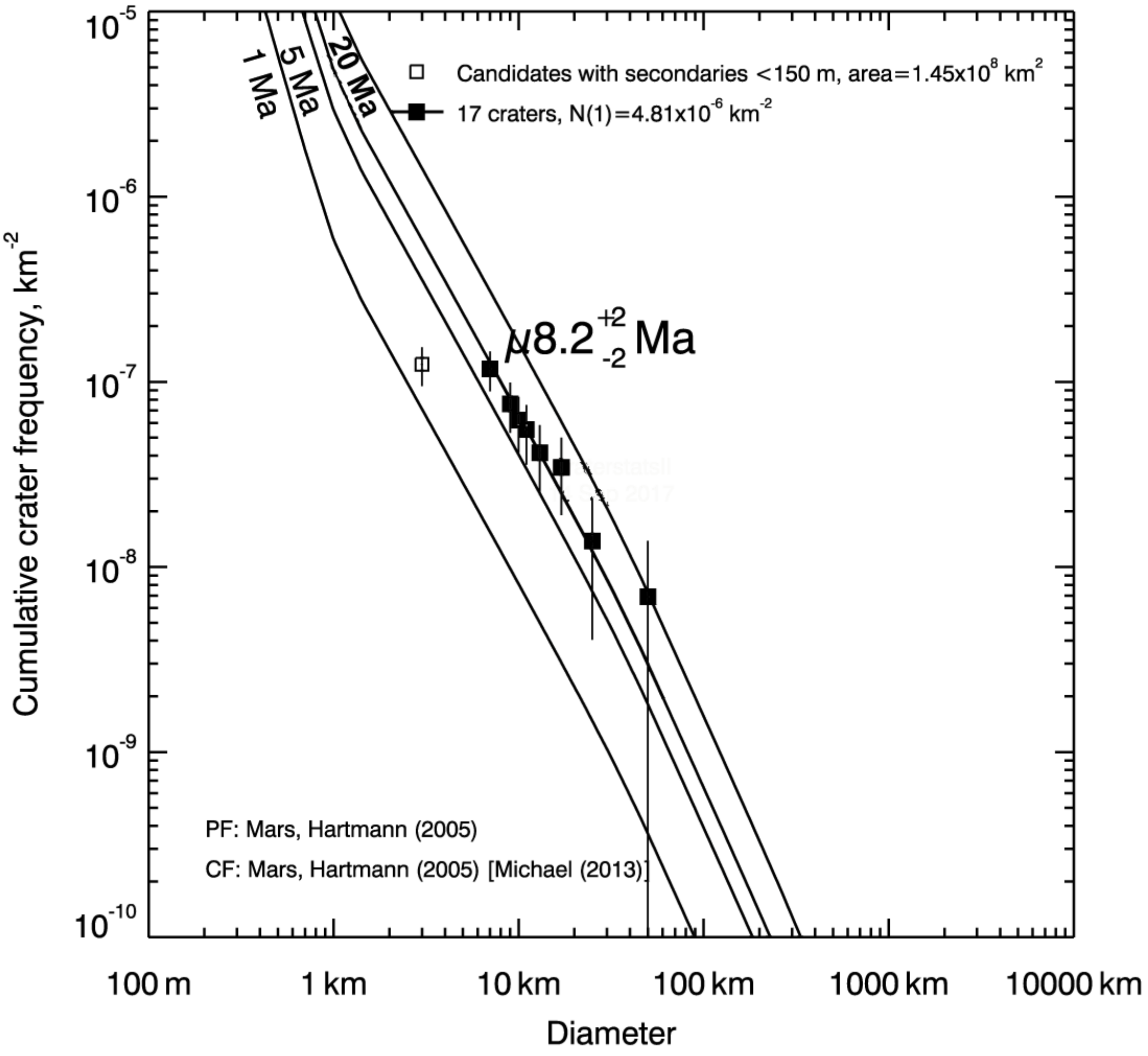

Supplementary Figure 6. Cumulative size-frequency distribution of the 18 craters identified in this study and younger than 10 Myr. Kotka crater is excluded as it is older (within error) than 10 Myr. The entire surface of Mars is used to compute the cumulative crater frequency. The distribution of craters larger than 7 km (17 craters over 18) defines an isochron at 8.2 Myr[3-5]. This fit indicates that our young large crater database can be considered complete for craters larger than 7 km in diameter. Error bars are generated from crater counts and dependant from the number of craters within each bin.

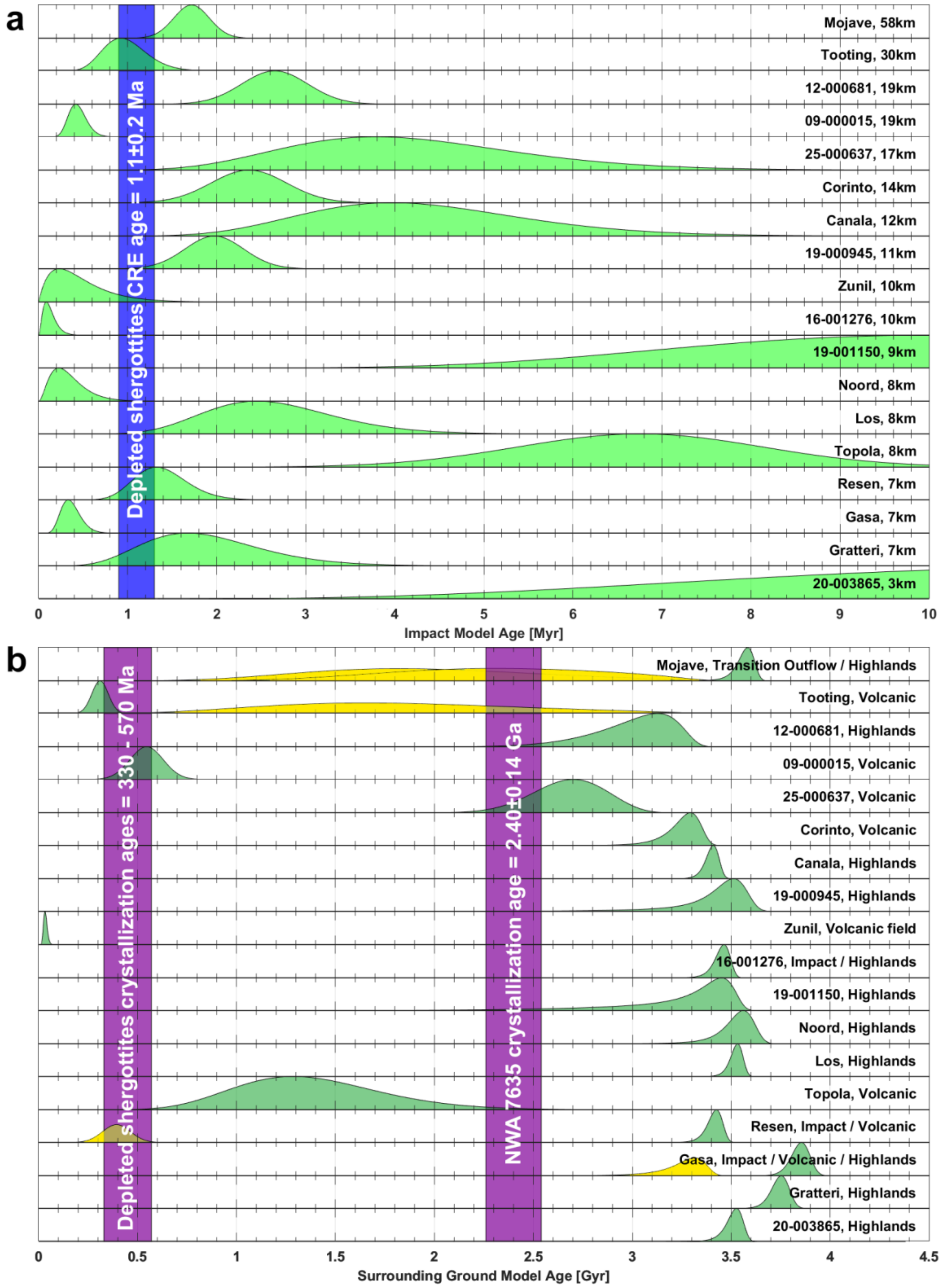

Supplementary Figure 7. Probability density function of model ages of crater candidates, and their surrounding ground. a: Crater model ages probability density function (in green) compared to the depleted shergottites CRE age[6,7] (in blue): 1.1±0.2 Ma. b: Surrounding ground model ages probability density function (in dark green) compared to the crystallization age range of depleted shergottites[6,7] (in purple): 330-570 Ma and 2.40±0.14 Ga (NWA 7635). Orange curves are resurfacing model ages (if younger than terrain hosts ages) or older ages derived from buried craters. All model ages probability density functions are derived from CraterStats II[3,4].

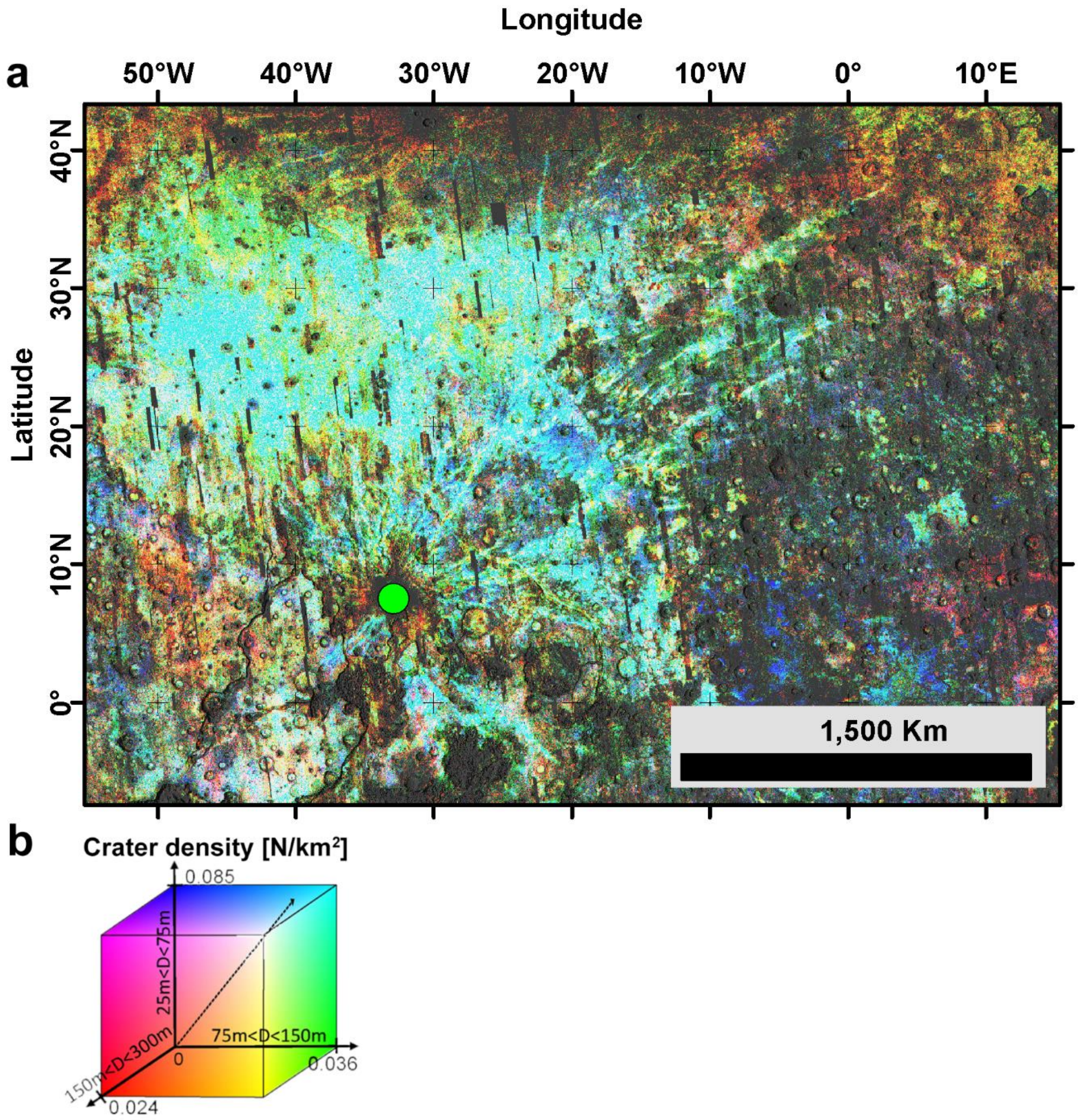

Supplementary Figure 8. Close-up view of the crater density map of Mojave crater. (a) The rays of secondary craters around Mojave (green circle) are visible in blue and green. (b) Legend of the crater density map shown on panel a. Each dimension of the colorcube corresponds to the crater density (number of craters per km^2) of specific diameter ranges (blue: 25 m – 75 m, green: 75 m - 150 m and red: 150 m – 300 m). Background: MOLA shaded relief (http://bit.ly/HRSC_MOLA_Blend_v0).

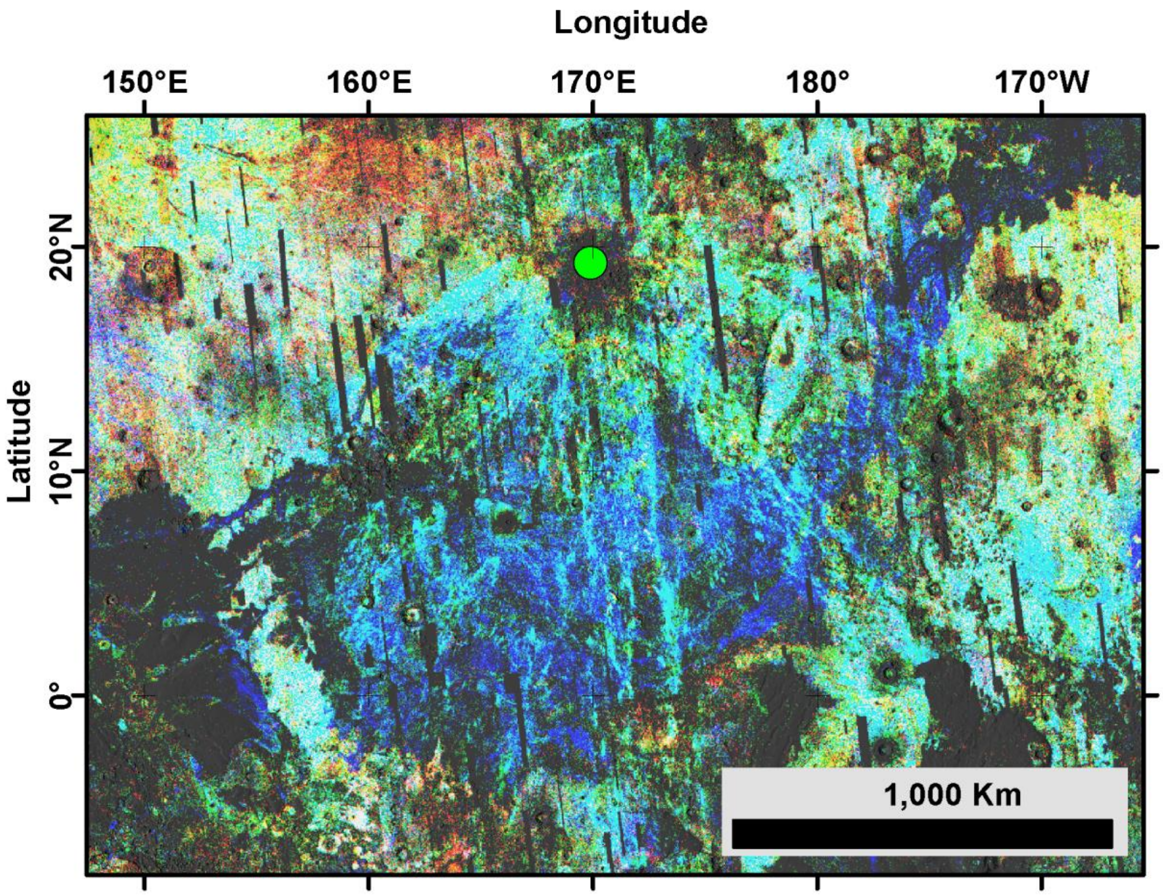

Supplementary Figure 9. Close-up view of the crater density map of Kotka crater. The rays of secondary craters around Kotka (green circle) are visible in blue and green. For the legend of the density map, readers are referred to the colorcube on Supplementary Figure 8.b. Background: MOLA shaded relief (http://bit.ly/HRSC_MOLA_Blend_v0).

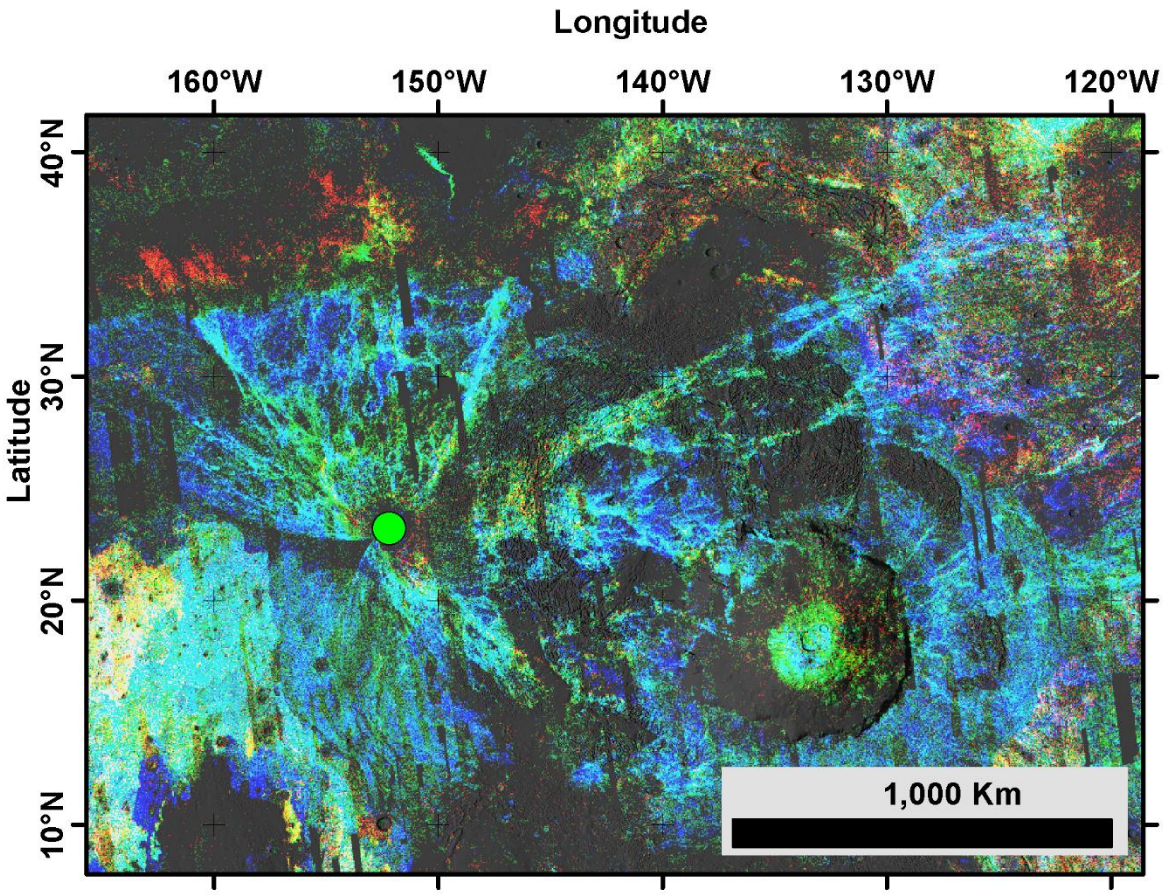

Supplementary Figure 10. Close-up view of the crater density map of Tooting crater. The rays of secondary craters around Tooting (green circle) are visible in blue and green. For the legend of the density map, readers are referred to the colorcube on Supplementary Figure 8.b. Background: MOLA shaded relief (http://bit.ly/HRSC_MOLA_Blend_v0).

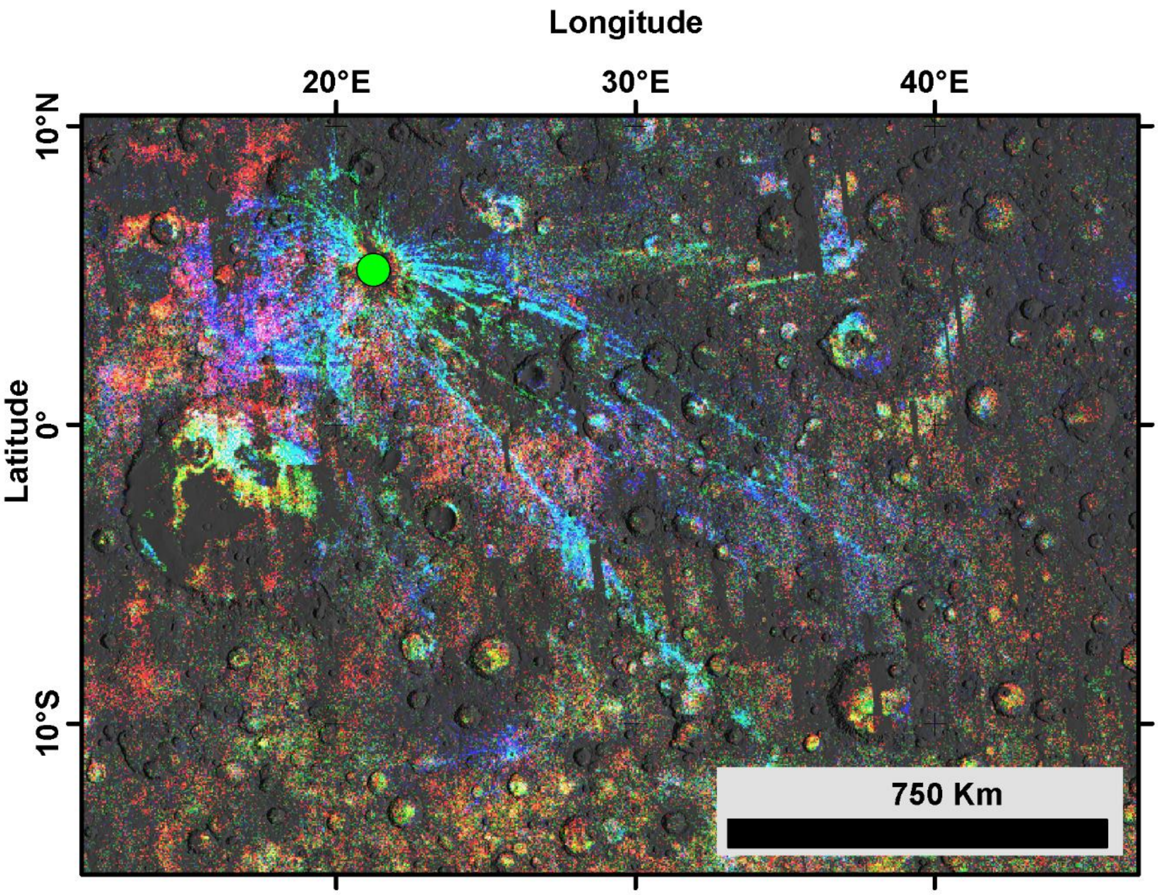

Supplementary Figure 11. Close-up view of the crater density map of 12-000681 crater. The rays of secondary craters around 12-000681 (green circle) are visible in blue and green. For the legend of the density map, readers are referred to the colorcube on Supplementary Figure 8.b. Background: MOLA shaded relief (http://bit.ly/HRSC_MOLA_Blend_v0).

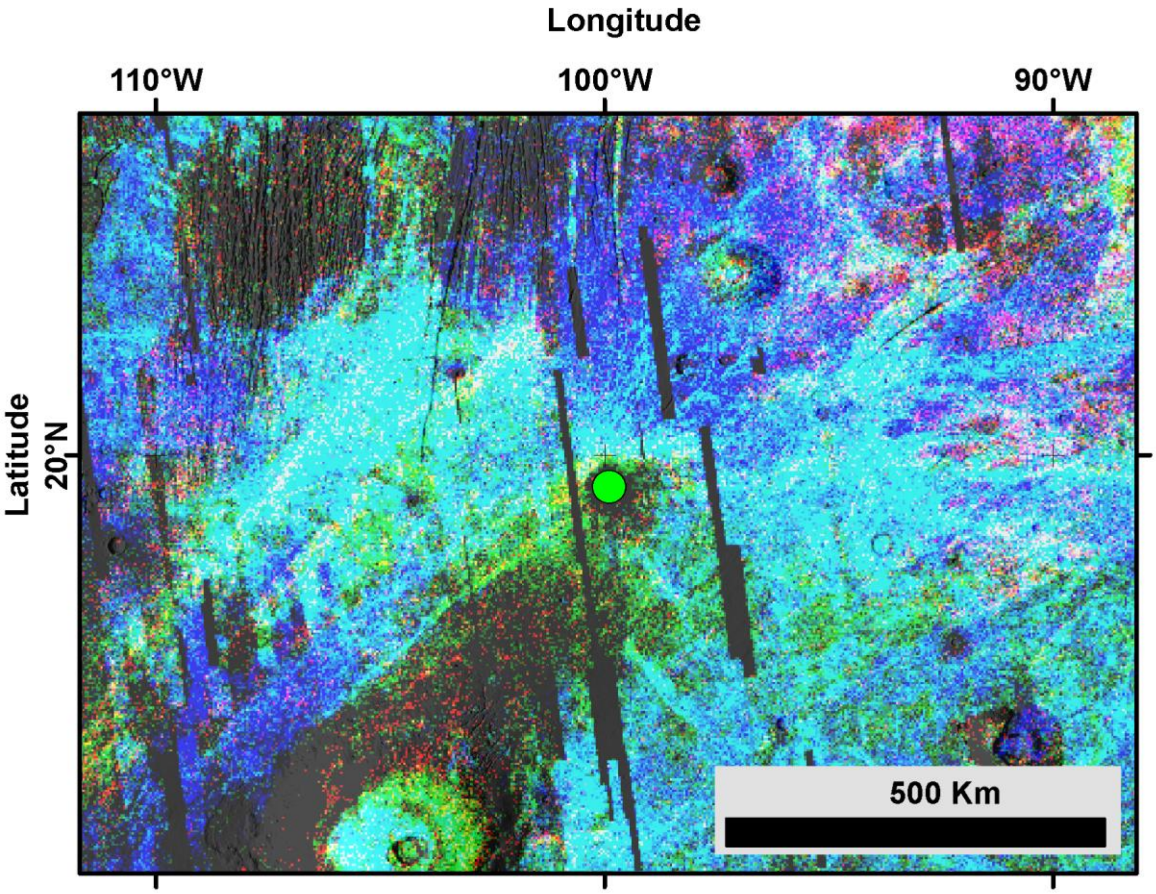

Supplementary Figure 12. Close-up view of the crater density map of 09-000015 crater. The rays of secondary craters around 09-000015 (green circle) are visible in blue and green. For the legend of the density map, readers are referred to the colorcube on Supplementary Figure 8.b. Background: MOLA shaded relief (http://bit.ly/HRSC_MOLA_Blend_v0).

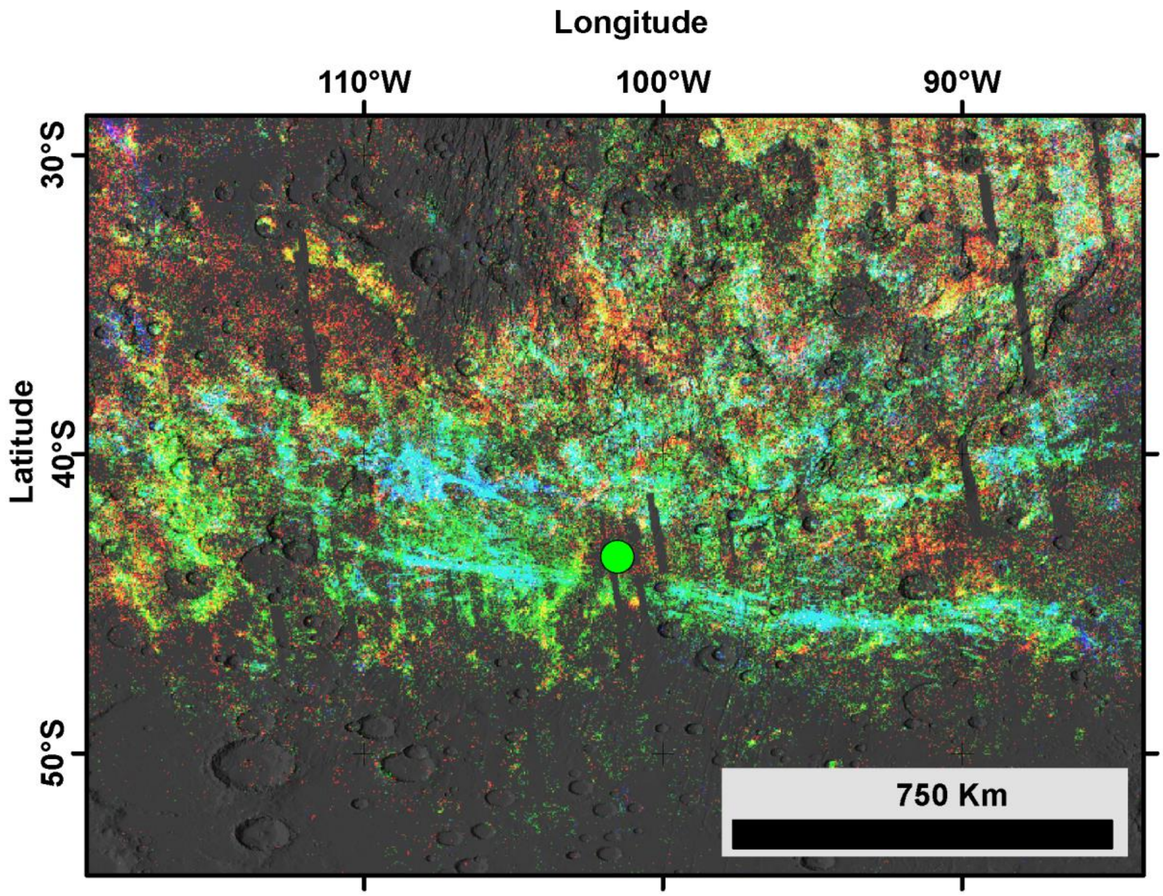

Supplementary Figure 13. Close-up view of the crater density map of 25-000637 crater. The rays of secondary craters around 25-000637 (green circle) are visible in blue and green. For the legend of the density map, readers are referred to the colorcube on Supplementary Figure 8.b. Background: MOLA shaded relief (http://bit.ly/HRSC_MOLA_Blend_v0).

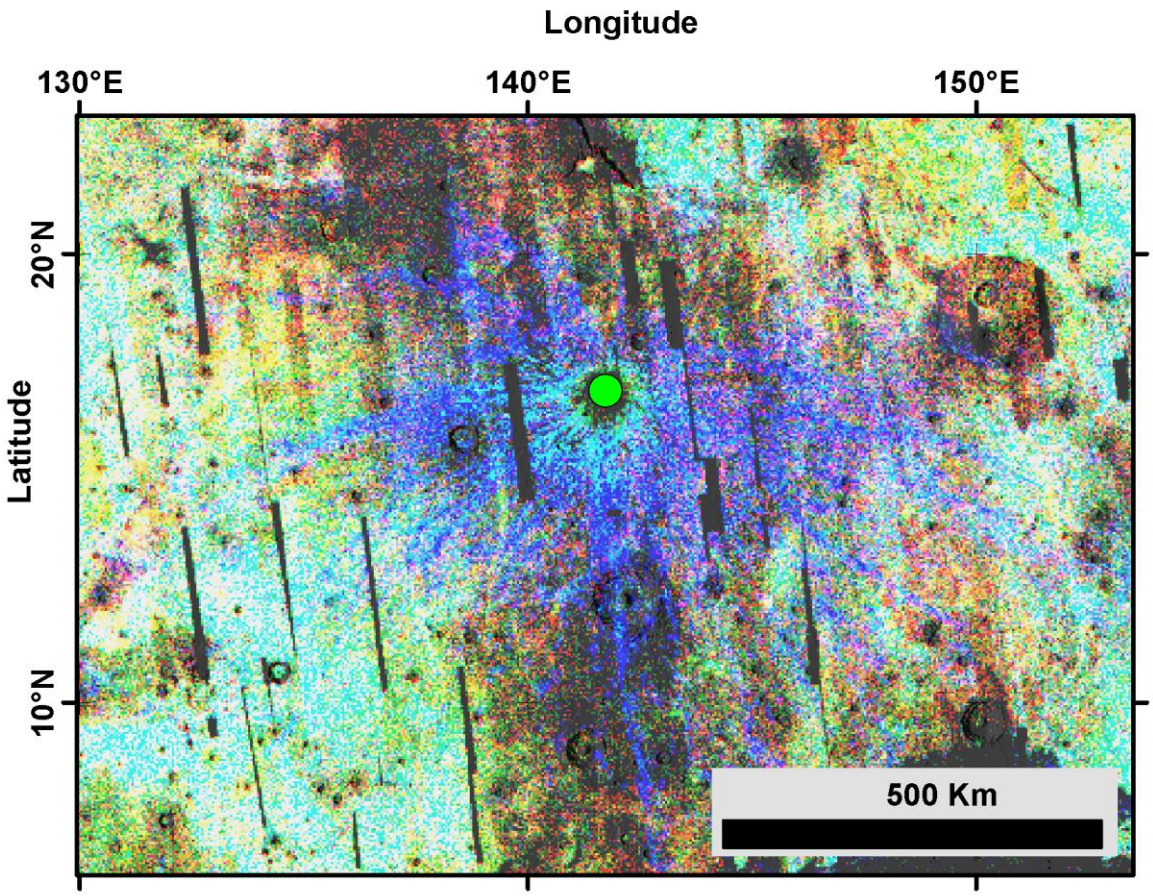

Supplementary Figure 14. Close-up view of the crater density map of Corinto crater. The rays of secondary craters around Corinto (green circle) are visible in blue and green. For the legend of the density map, readers are referred to the colorcube on Supplementary Figure 8.b. Background: MOLA shaded relief (http://bit.ly/HRSC_MOLA_Blend_v0).

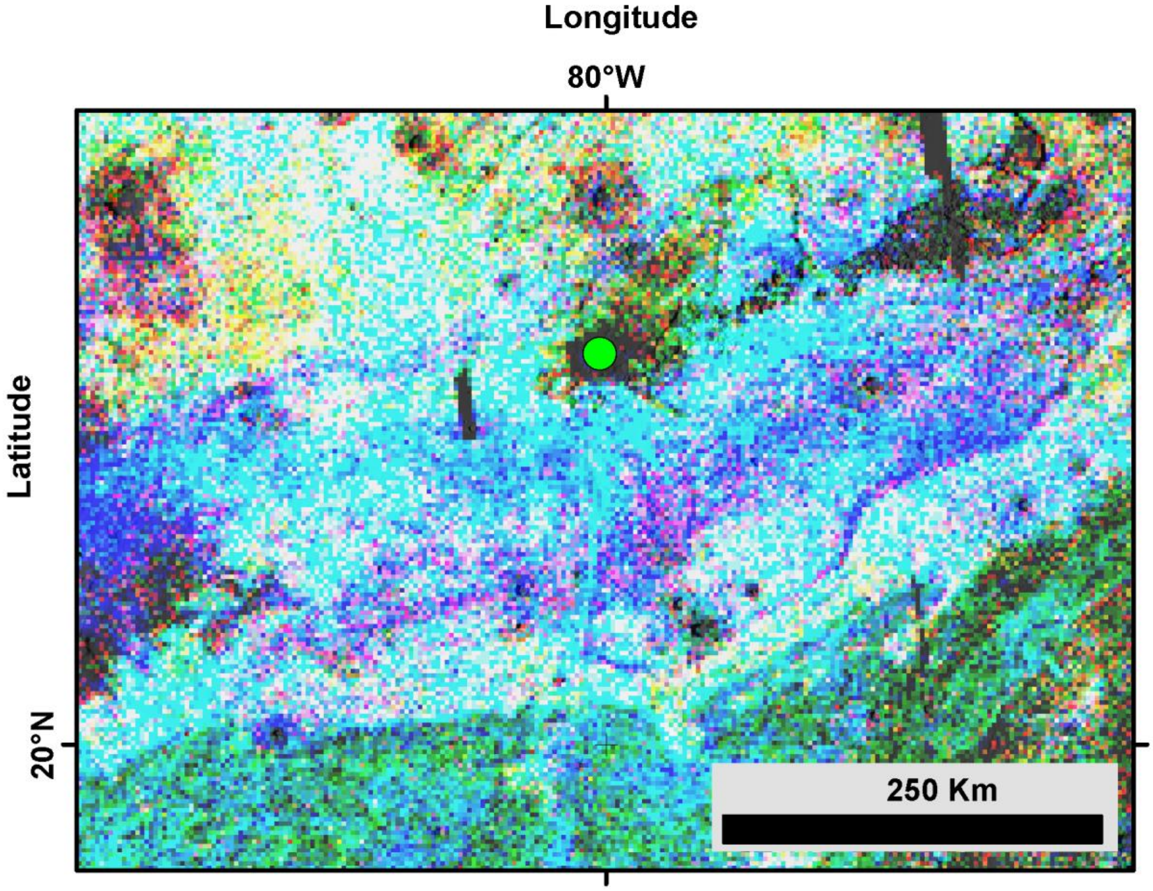

Supplementary Figure 15. Close-up view of the crater density map of Canala crater. The rays of secondary craters around Canala (green circle) are visible in blue and green. For the legend of the density map, readers are referred to the colorcube on Supplementary Figure 8.b. Background: MOLA shaded relief (http://bit.ly/HRSC_MOLA_Blend_v0).

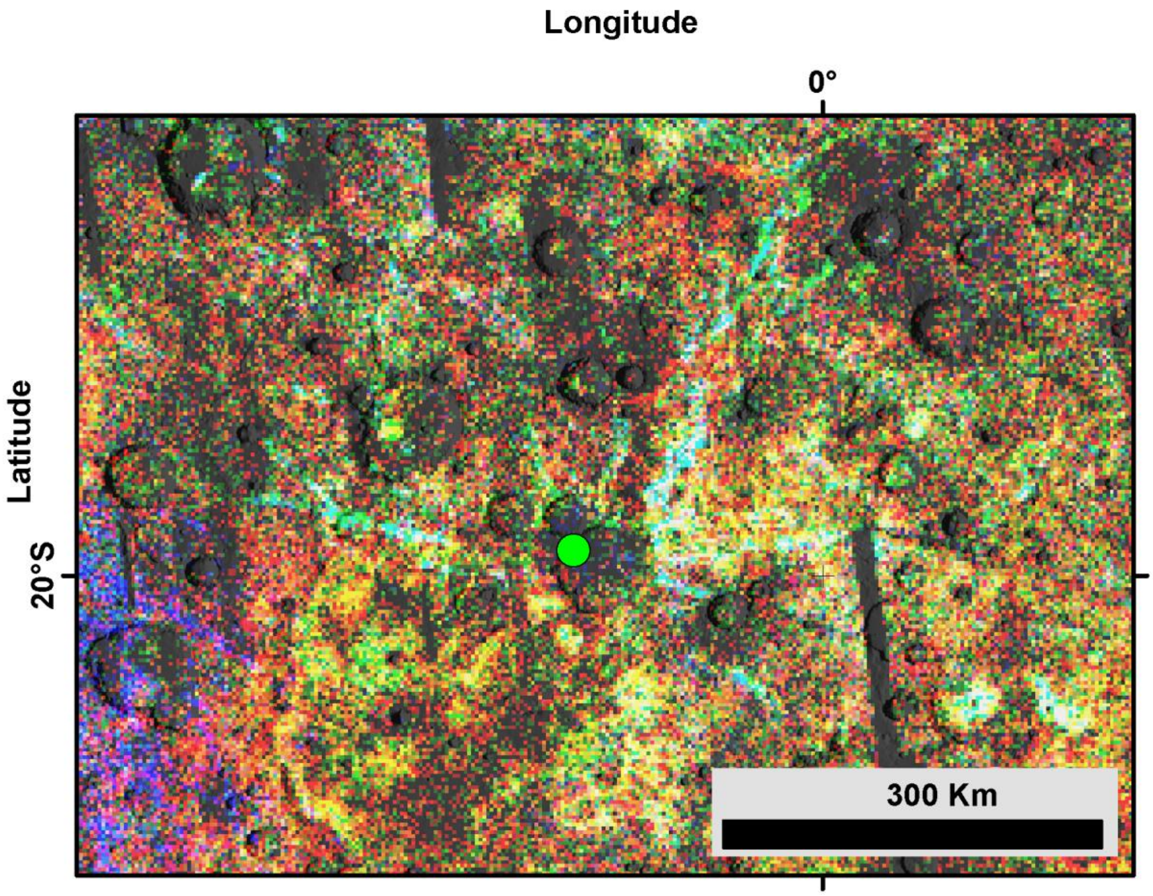

Supplementary Figure 16. Close-up view of the crater density map of 19-000945 crater. The rays of secondary craters around 19-000945 (green circle) are visible in blue and green. For the legend of the density map, readers are referred to the colorcube on Supplementary Figure 8.b. Background: MOLA shaded relief (http://bit.ly/HRSC_MOLA_Blend_v0).

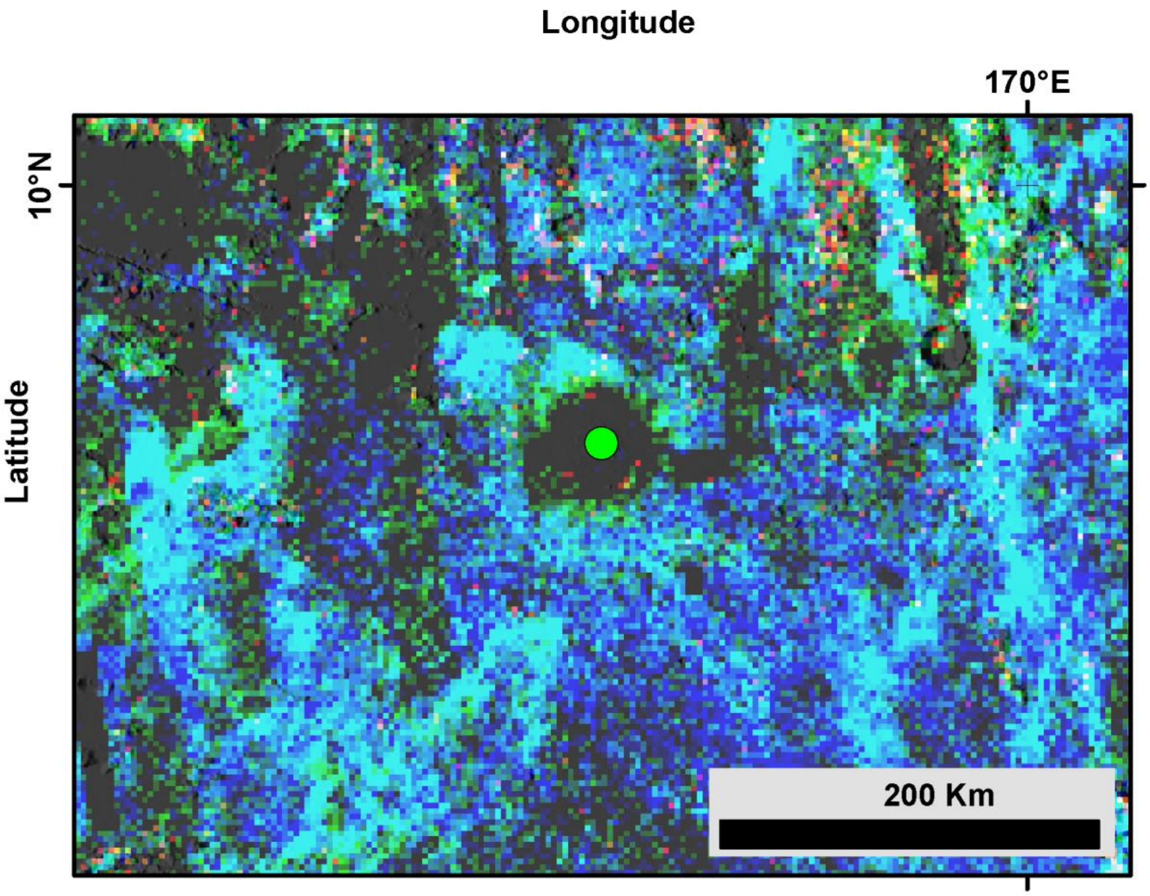

Supplementary Figure 17. Close-up view of the crater density map of Zunil crater. The rays of secondary craters around Zunil (green circle) are visible in blue and green. For the legend of the density map, readers are referred to the colorcube on Supplementary Figure 8.b. Background: MOLA shaded relief (http://bit.ly/HRSC_MOLA_Blend_v0).

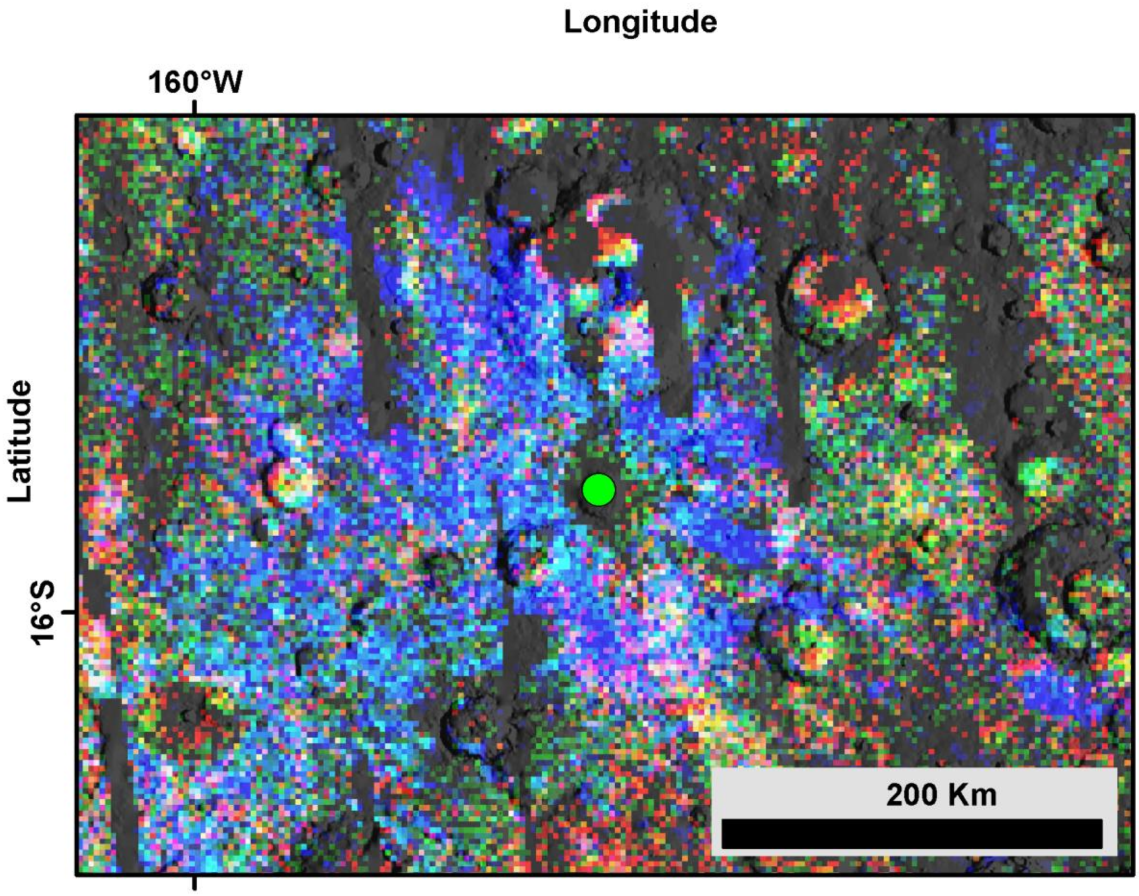

Supplementary Figure 18. Close-up view of the crater density map of 16-001276 crater. The rays of secondary craters around 16-001276 (green circle) are visible in blue and green. For the legend of the density map, readers are referred to the colorcube on Supplementary Figure 8.b. Background: MOLA shaded relief (http://bit.ly/HRSC_MOLA_Blend_v0).

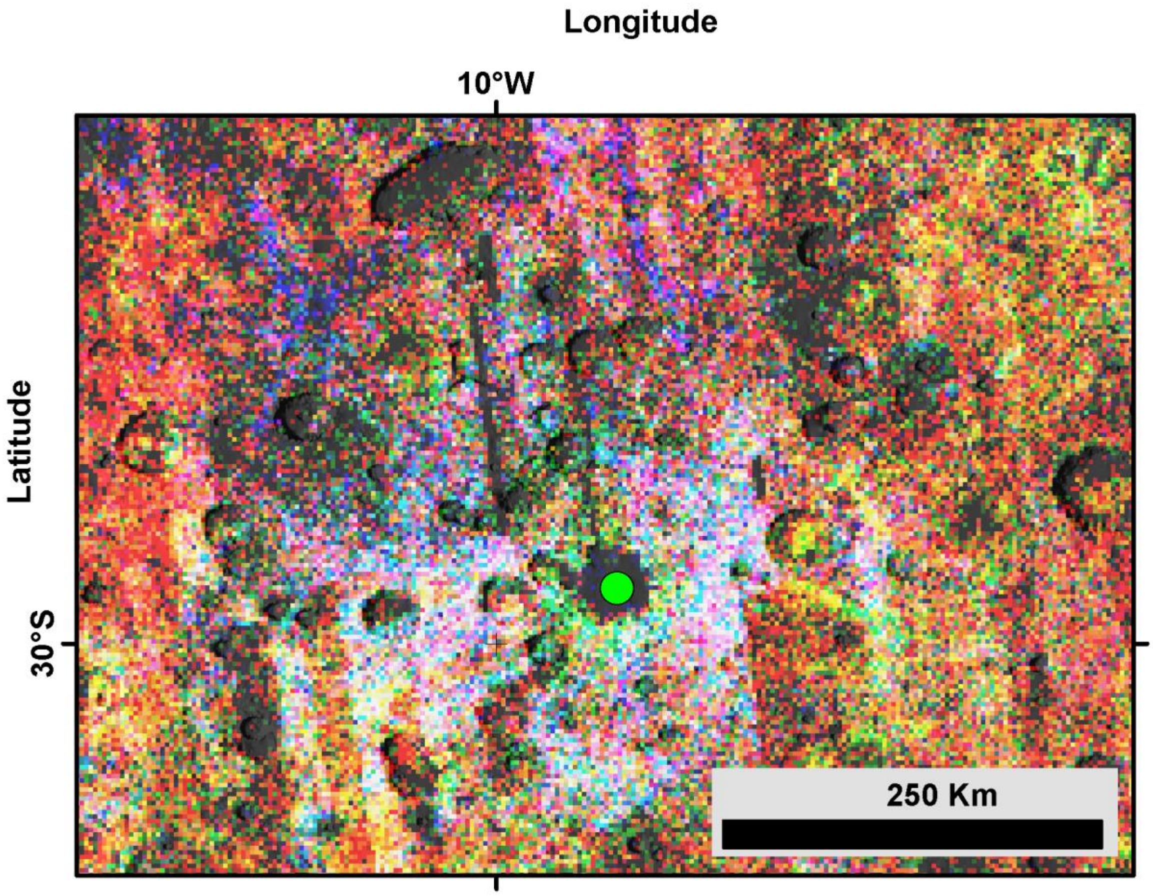

Supplementary Figure 19. Close-up view of the crater density map of 19-01150 crater. The rays of secondary craters around 19-01150 (green circle) are visible in blue and green. For the legend of the density map, readers are referred to the colorcube on Supplementary Figure 8.b. Background: MOLA shaded relief (http://bit.ly/HRSC_MOLA_Blend_v0).

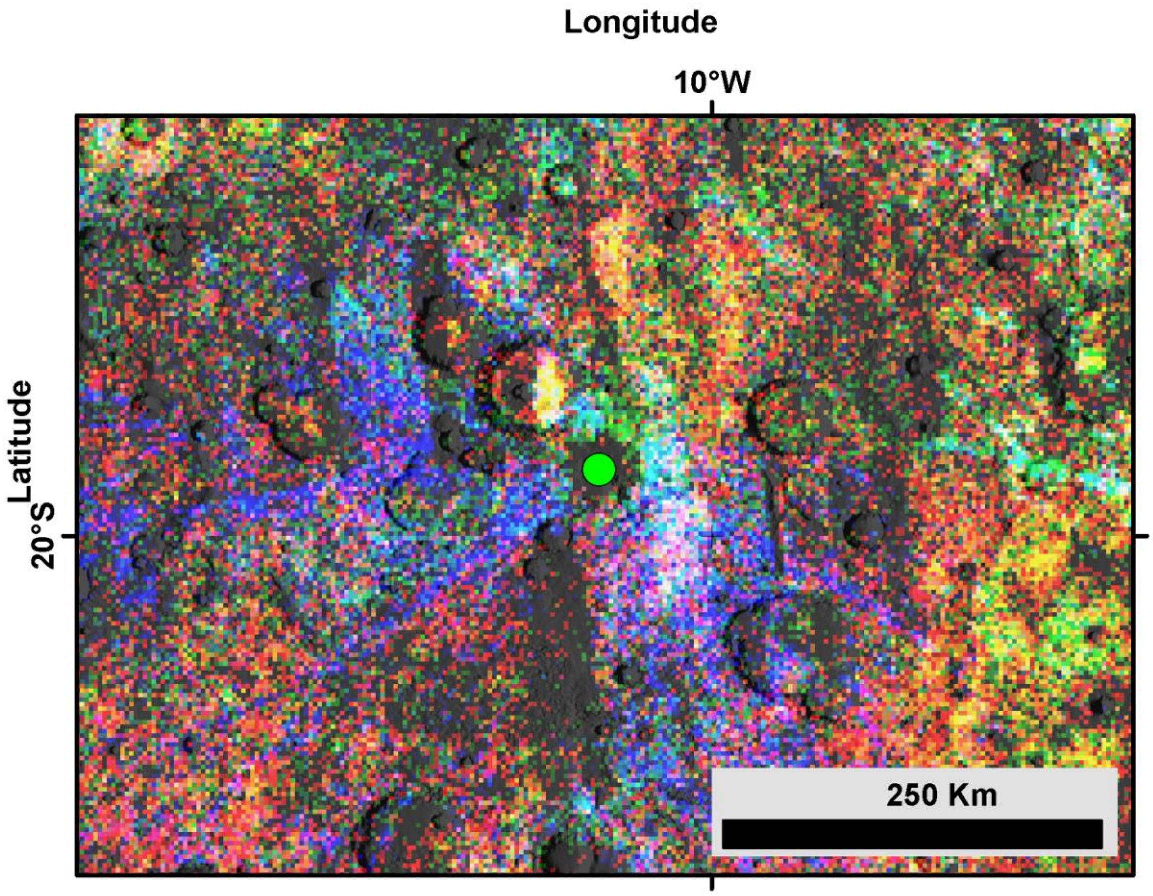

Supplementary Figure 20. Close-up view of the crater density map of Noord crater. The rays of secondary craters around Noord (green circle) are visible in blue and green. For the legend of the density map, readers are referred to the colorcube on Supplementary Figure 8.b. Background: MOLA shaded relief (http://bit.ly/HRSC_MOLA_Blend_v0).

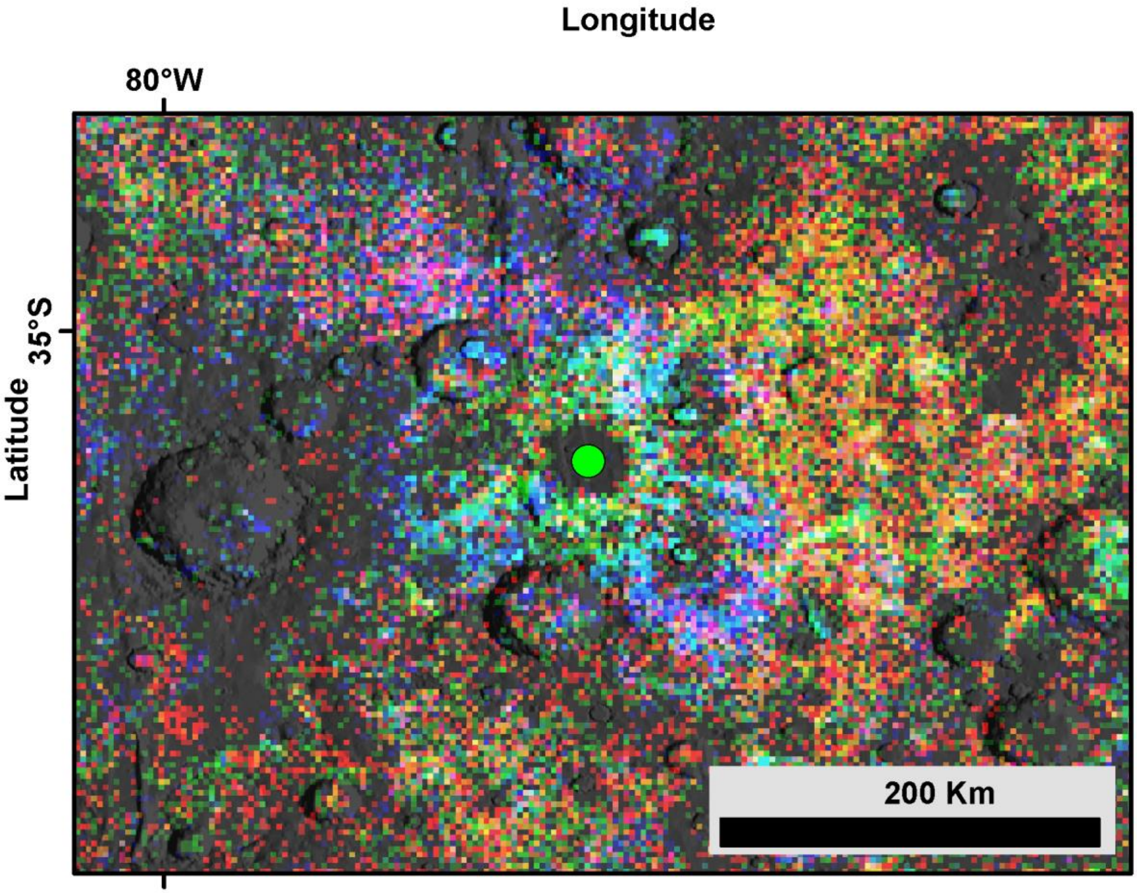

Supplementary Figure 21. Close-up view of the crater density map of Los crater. The rays of secondary craters around Los (green circle) are visible in blue and green. For the legend of the density map, readers are referred to the colorcube on Supplementary Figure 8.b. Background: MOLA shaded relief (http://bit.ly/HRSC_MOLA_Blend_v0).

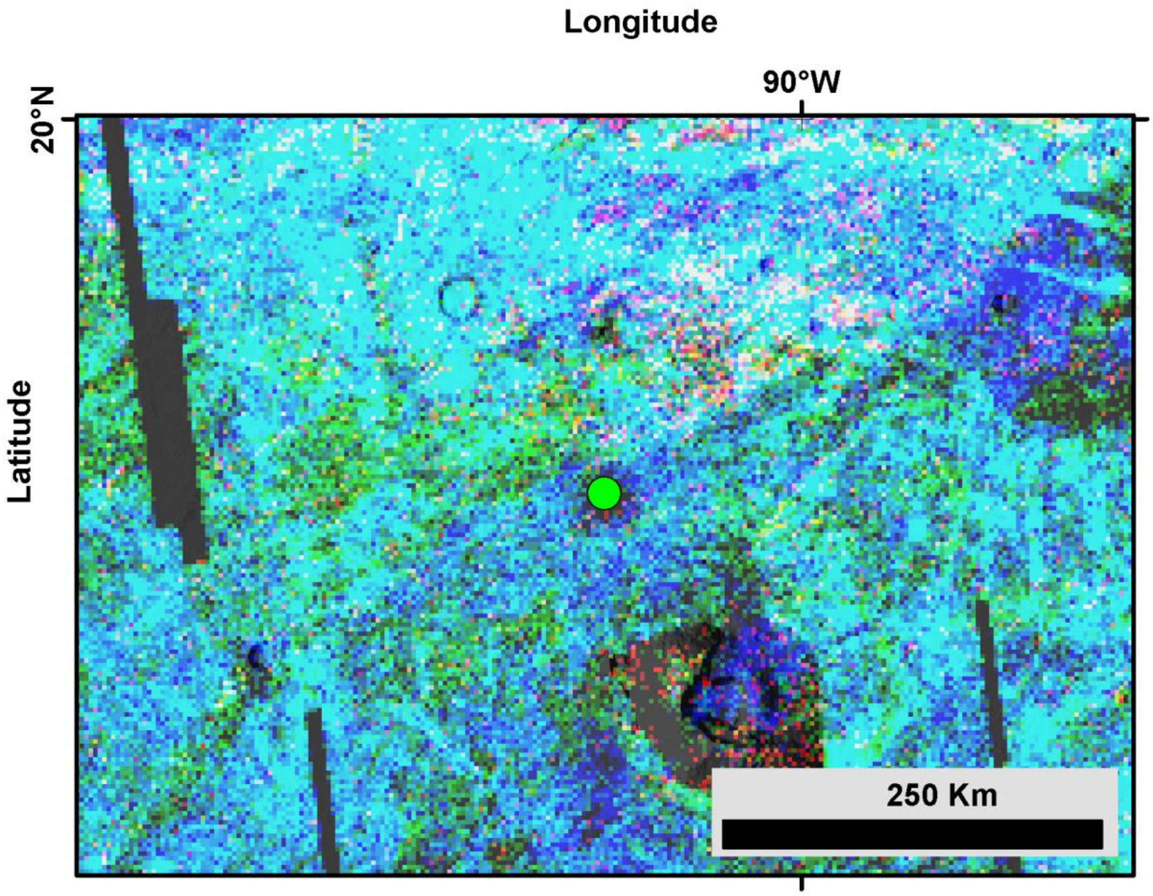

Supplementary Figure 22. Close-up view of the crater density map of Topola crater. The rays of secondary craters around Topola (green circle) are visible in blue and green. For the legend of the density map, readers are referred to the colorcube on Supplementary Figure 8.b. Background: MOLA shaded relief (http://bit.ly/HRSC_MOLA_Blend_v0).

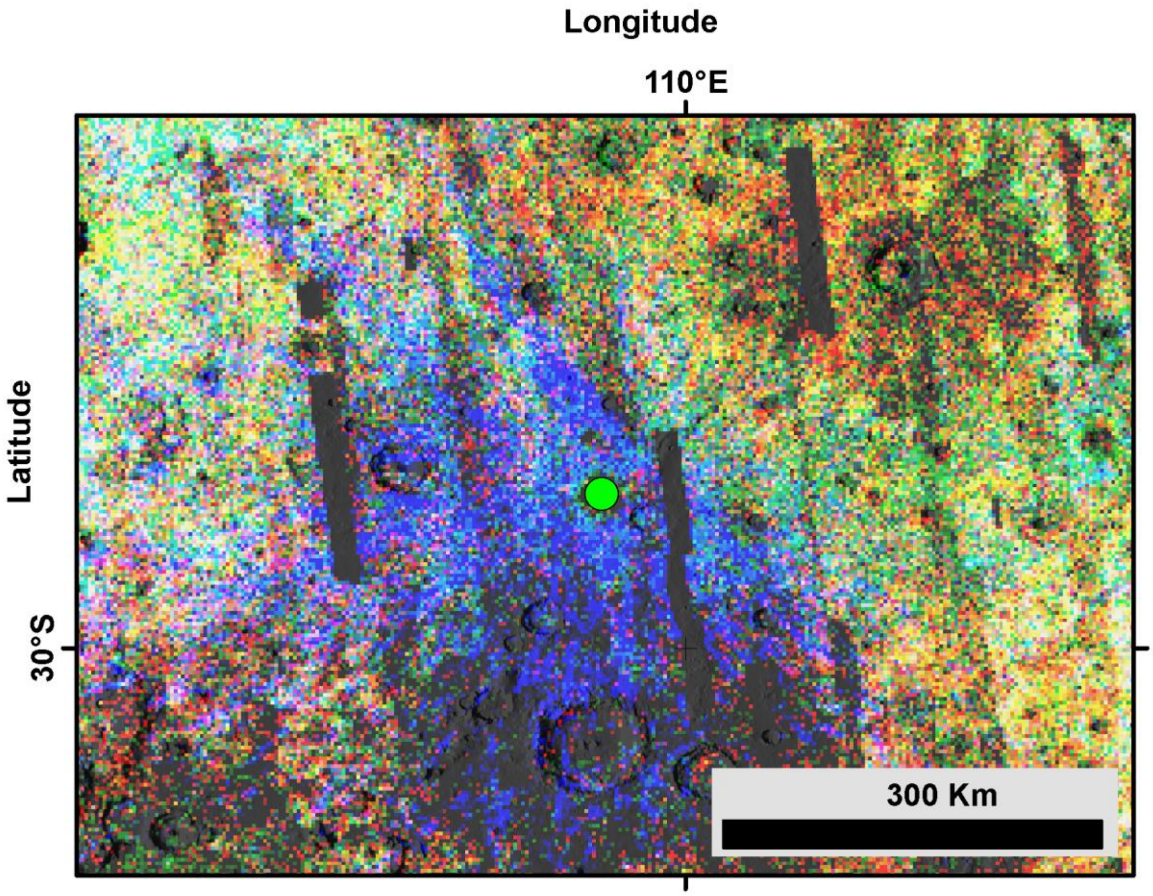

Supplementary Figure 23. Close-up view of the crater density map of Resen crater. The rays of secondary craters around Resen (green circle) are visible in blue and green. For the legend of the density map, readers are referred to the colorcube on Supplementary Figure 8.b. Background: MOLA shaded relief (http://bit.ly/HRSC_MOLA_Blend_v0).

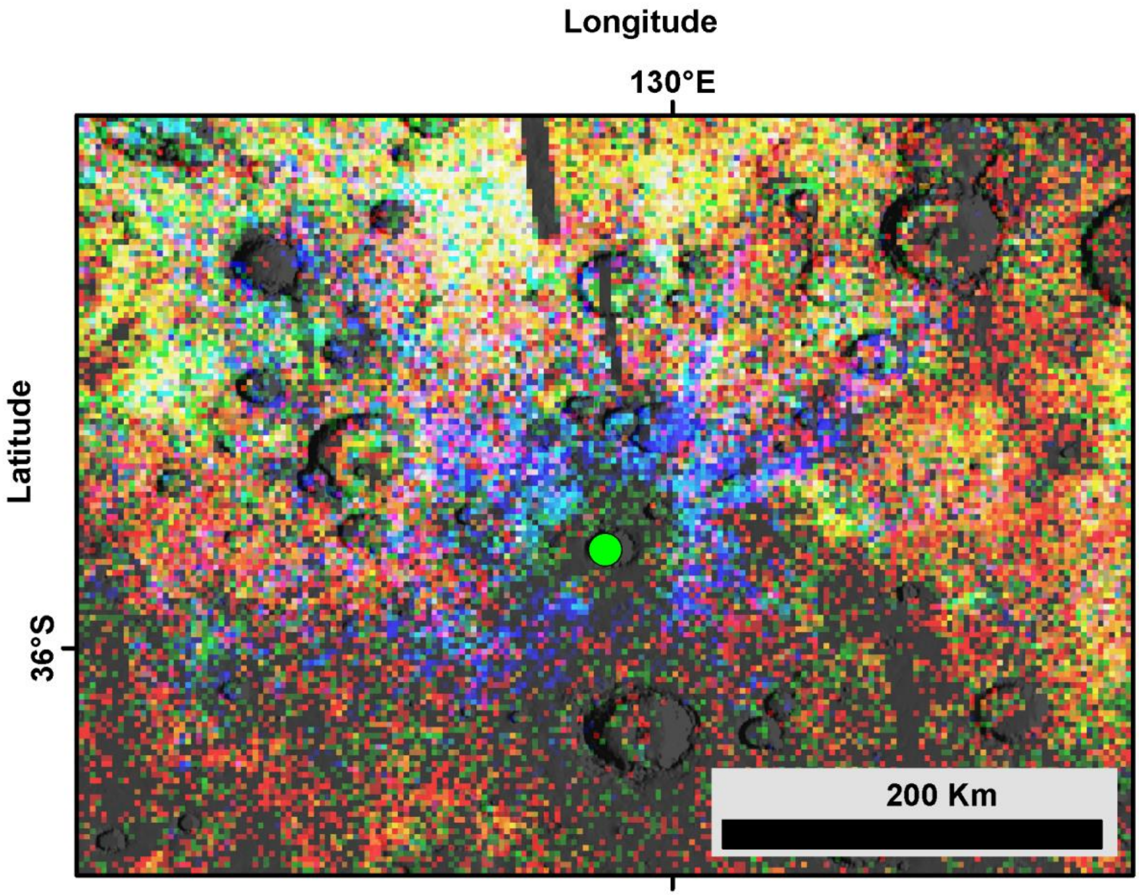

Supplementary Figure 24. Close-up view of the crater density map of Gasa crater. The rays of secondary craters around Gasa (green circle) are visible in blue and green. For the legend of the density map, readers are referred to the colorcube on Supplementary Figure 8.b. Background: MOLA shaded relief (http://bit.ly/HRSC_MOLA_Blend_v0).

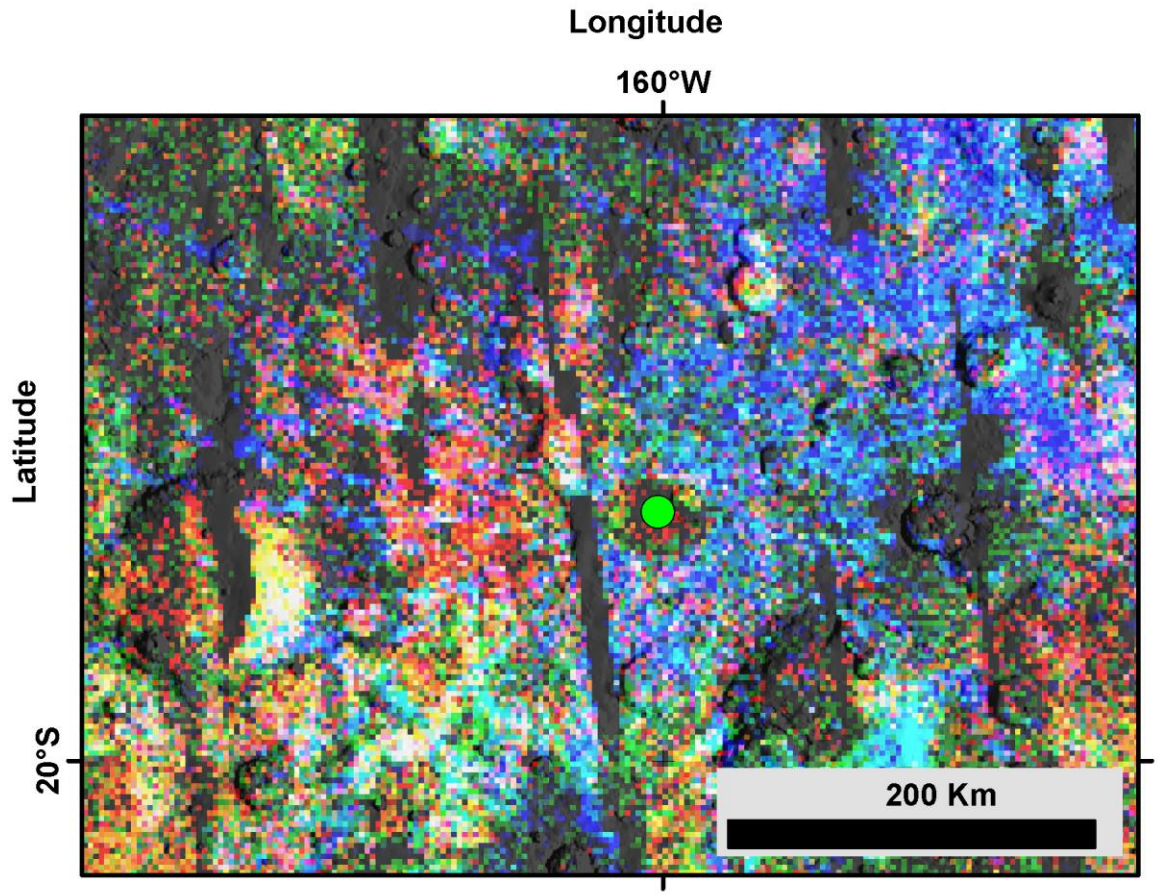

Supplementary Figure 25. Close-up view of the crater density map of Gratteri crater. The rays of secondary craters around Gratteri (green circle) are visible in blue and green. For the legend of the density map, readers are referred to the colorcube on Supplementary Figure 8.b. Background: MOLA shaded relief (http://bit.ly/HRSC_MOLA_Blend_v0).

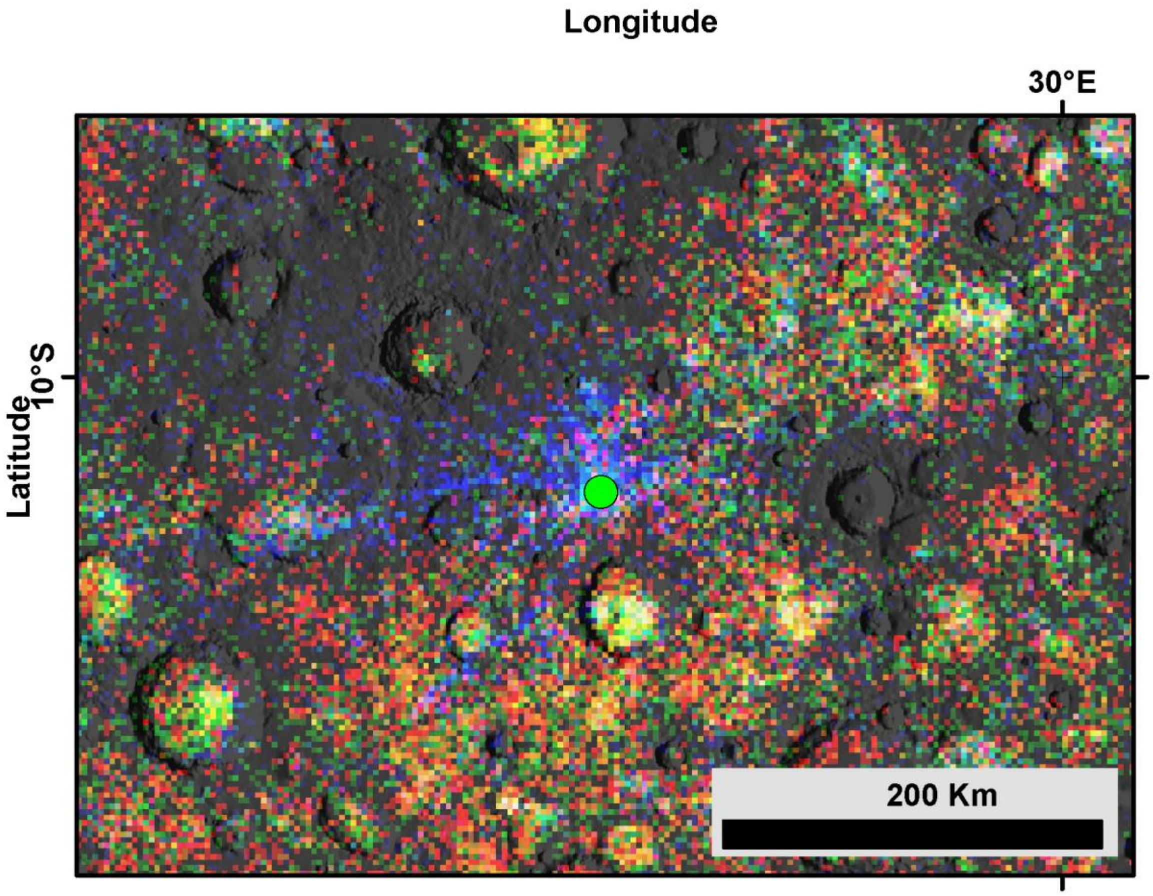

Supplementary Figure 26. Close-up view of the crater density map of 20-003865 crater. The rays of secondary craters around 20-003865 (green circle) are visible in blue and green. For the legend of the density map, readers are referred to the colorcube on Supplementary Figure 8.b. Background: MOLA shaded relief (http://bit.ly/HRSC_MOLA_Blend_v0).

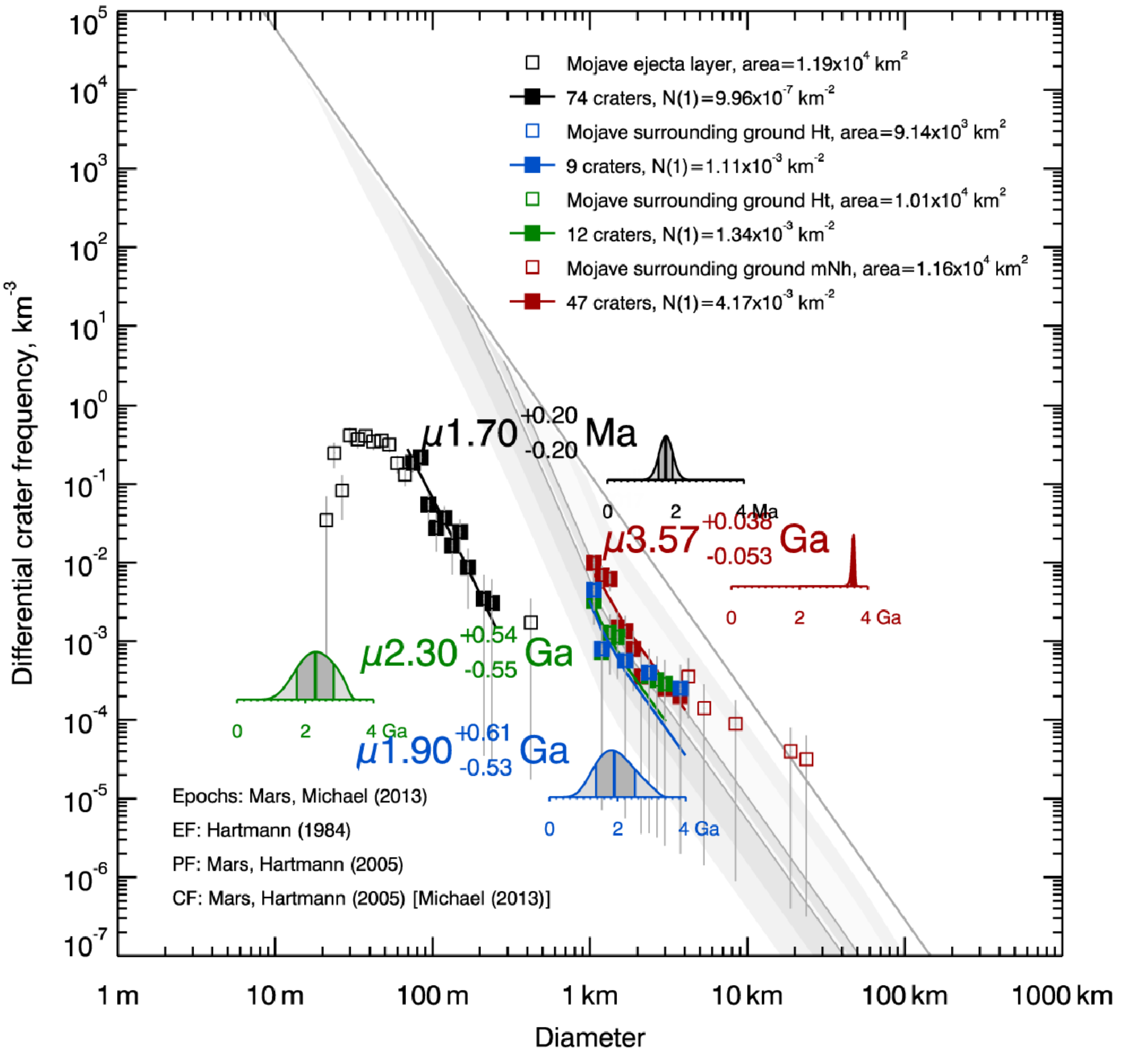

Supplementary Figure 27. Crater count and model age derivation results of Mojave crater. Black CSFD corresponds to craters superposed on the ejecta blanket. The red CSFD is obtained from craters superposed to the immediate vicinity of the surrounding terrain, on the mNh (middle Noachian highlands) unit, while the blue and the green CSFDs correspond respectively to crater counts performed on two distinct Ht (Hesperian transition) units[8]. CSFDs are displayed as differential crater frequency versus diameter[9,10]. The error bars are dependent of the number of craters counted within each diameter bin. The Poisson timing technique has been used to fit an isochrone with the observed CSFD[4]. Note that this fitting technique is independent of the binning technique used to compute the CSFD[3]. An artificial discrepancy can therefore be observed in some cases but do not result in a misinterpretation of the data[4]. Grey isochones correspond to the limit between martian epochs, from the left to the right: late Amazonian – middle Amazonian, middle Noachian – early Noachian[3,5].

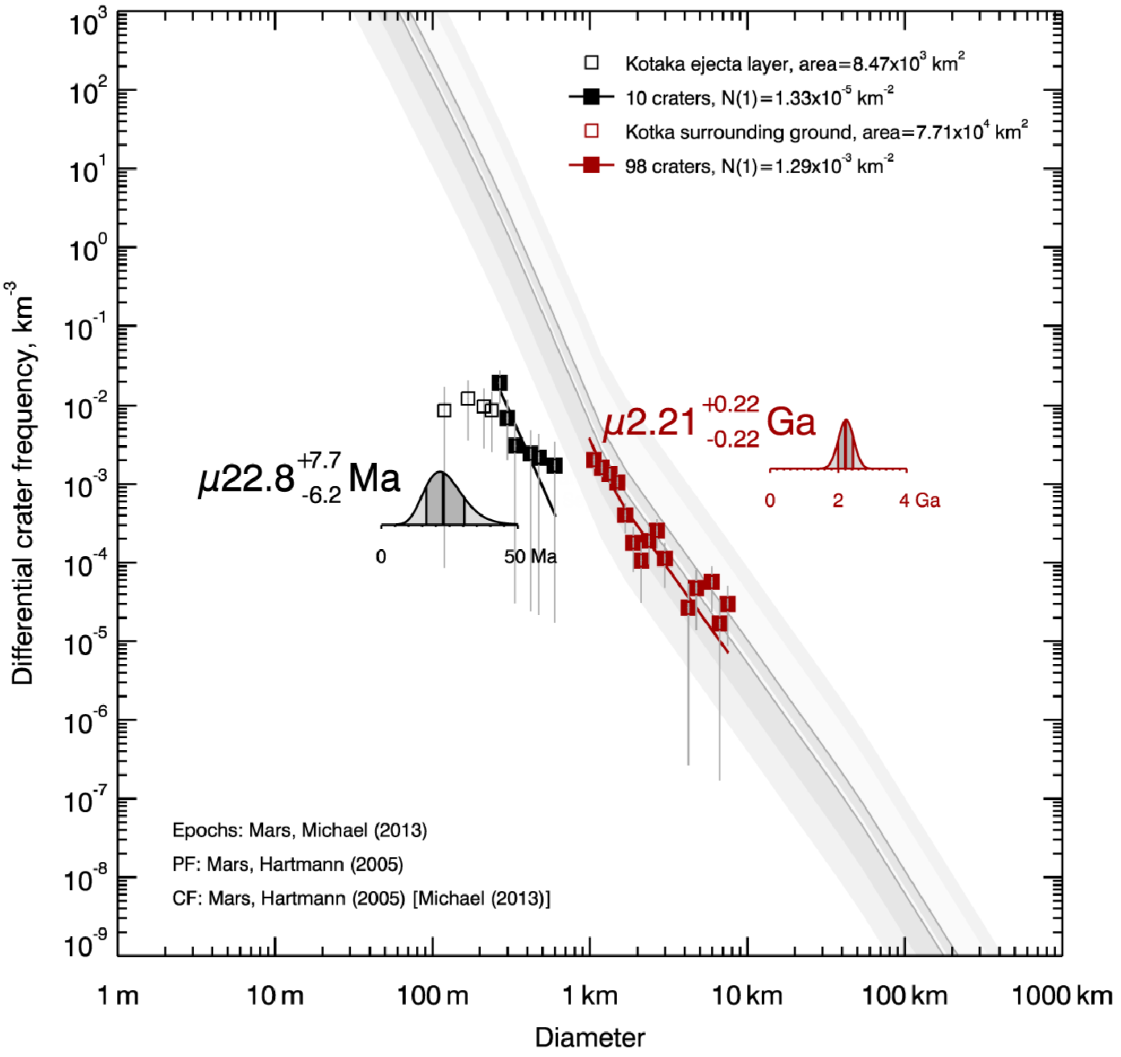

Supplementary Figure 28. Crater count and model age derivation results of Kotka crater. Black CSFD corresponds to craters superposed on the ejecta blanket. The red CSFD is obtained from craters superposed to the immediate vicinity of its surrounding terrain. CSFDs are displayed as differential crater frequency versus diameter[9,10]. The error bars are dependent of the number of craters counted within each diameter bin. The Poisson timing technique has been used to fit an isochrone with the observed CSFD[4]. Note that this fitting technique is independent of the binning technique used to compute the CSFD[3]. An artificial discrepancy can therefore be observed in some cases but do not result in a misinterpretation of the data[4]. Grey isochones correspond to the limit between martian epochs, from the left to the right: late Amazonian – middle Amazonian, middle Noachian – early Noachian[3,5].

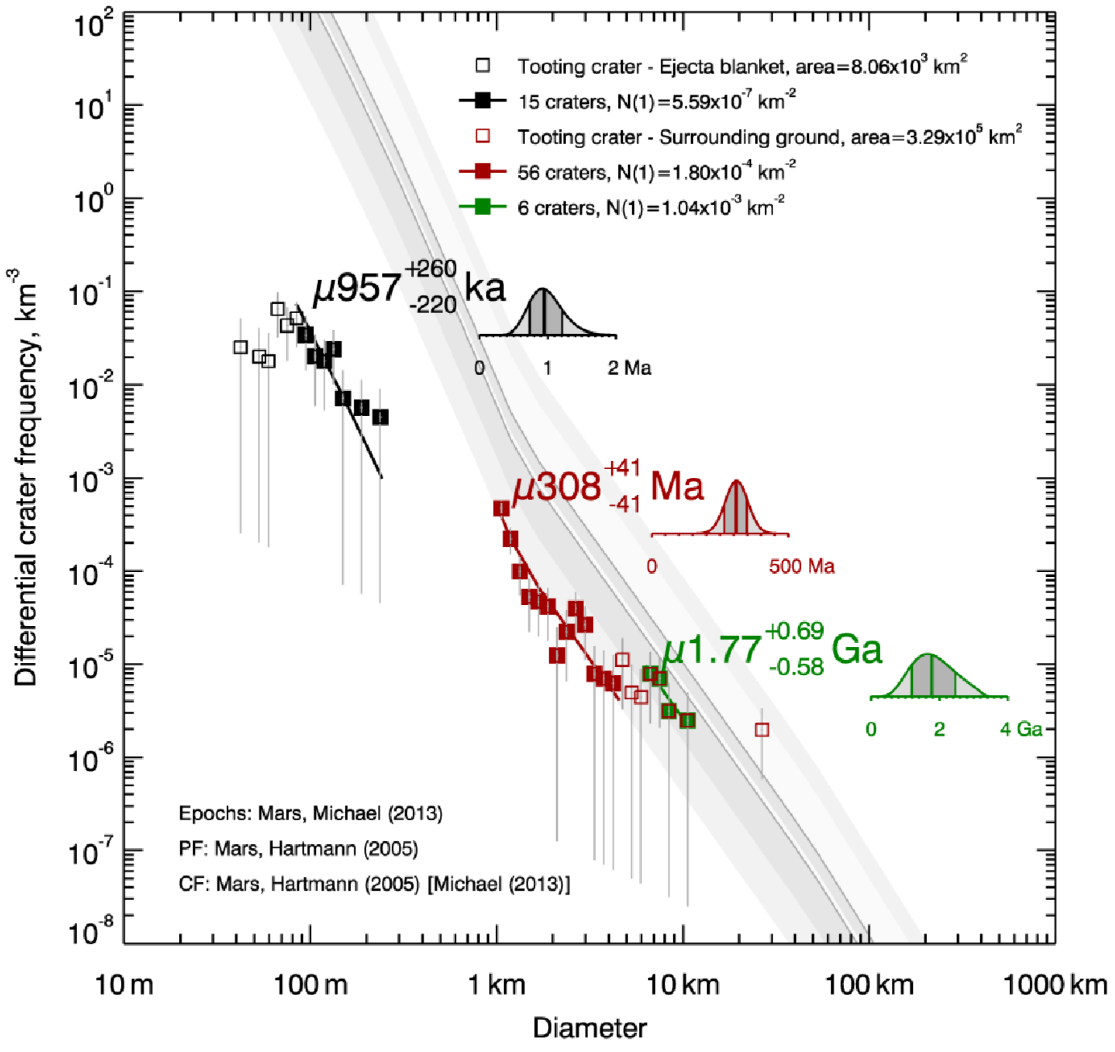

Supplementary Figure 29. Crater count and model age derivation results of Tooting crater. Black CSFD corresponds to craters superposed on the ejecta blanket. The red CSFD is obtained from craters superposed to the immediate vicinity of its surrounding terrain, while the green CSFD corresponds to highly degraded craters or infilled by recent volcanic deposit. CSFDs are displayed as differential crater frequency versus diameter[9,10]. The error bars are dependent of the number of craters counted within each diameter bin. The Poisson timing technique has been used to fit an isochrone with the observed CSFD[4]. Note that this fitting technique is independent of the binning technique used to compute the CSFD[3]. An artificial discrepancy can therefore be observed in some cases but do not result in a misinterpretation of the data[4]. Grey isochones correspond to the limit between martian epochs, from the left to the right: late Amazonian – middle Amazonian, middle Noachian – early Noachian[3,5].

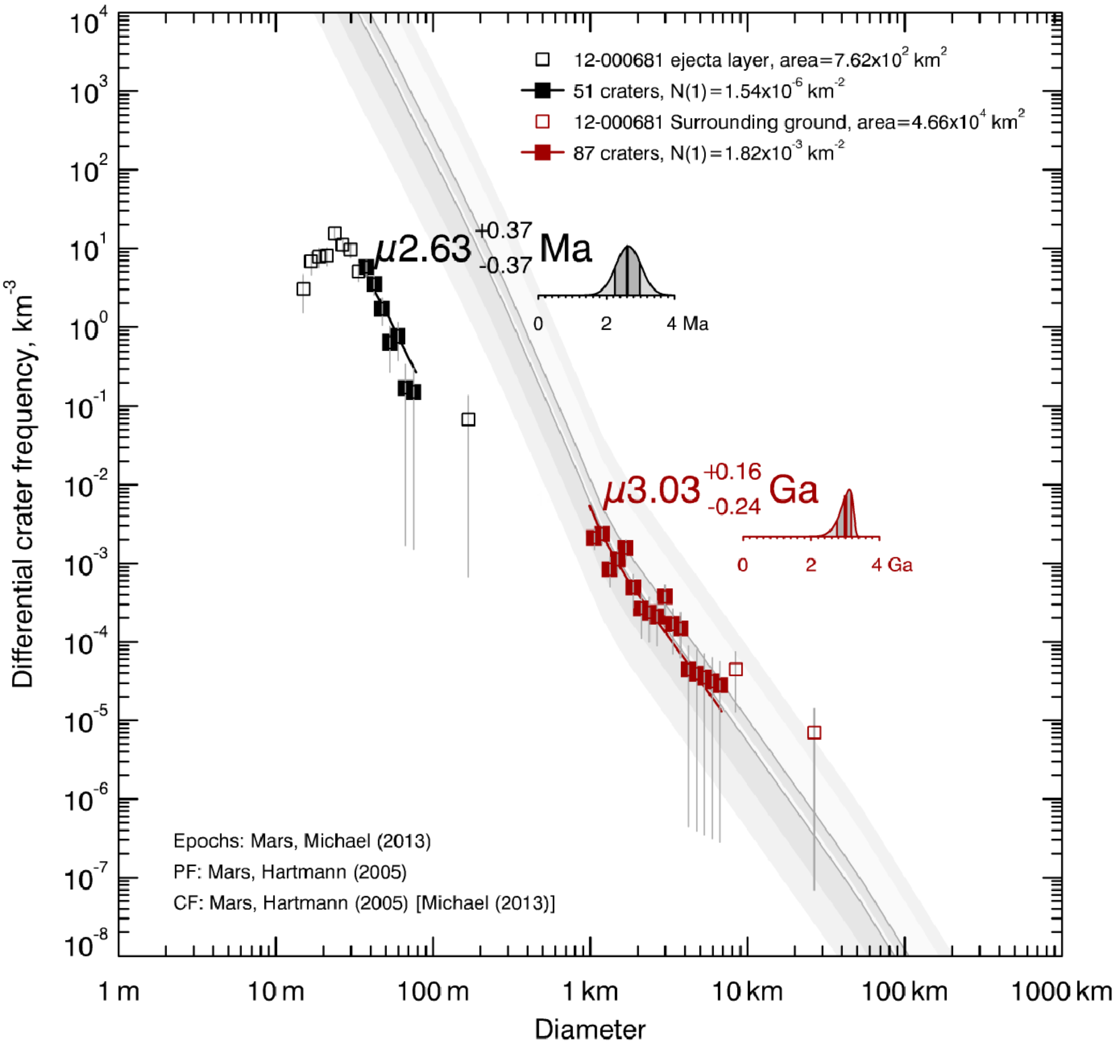

Supplementary Figure 30. Crater count and model age derivation results of 12-000681 crater. Black CSFD corresponds to craters superposed on the ejecta blanket. The red CSFD is obtained from craters superposed to the immediate vicinity of its surrounding terrain. CSFDs are displayed as differential crater frequency versus diameter[9,10]. The error bars are dependent of the number of craters counted within each diameter bin. The Poisson timing technique has been used to fit an isochrone with the observed CSFD[4]. Note that this fitting technique is independent of the binning technique used to compute the CSFD[3]. An artificial discrepancy can therefore be observed in some cases but do not result in a misinterpretation of the data[4]. Grey isochones correspond to the limit between martian epochs, from the left to the right: late Amazonian – middle Amazonian, middle Noachian – early Noachian[3,5].

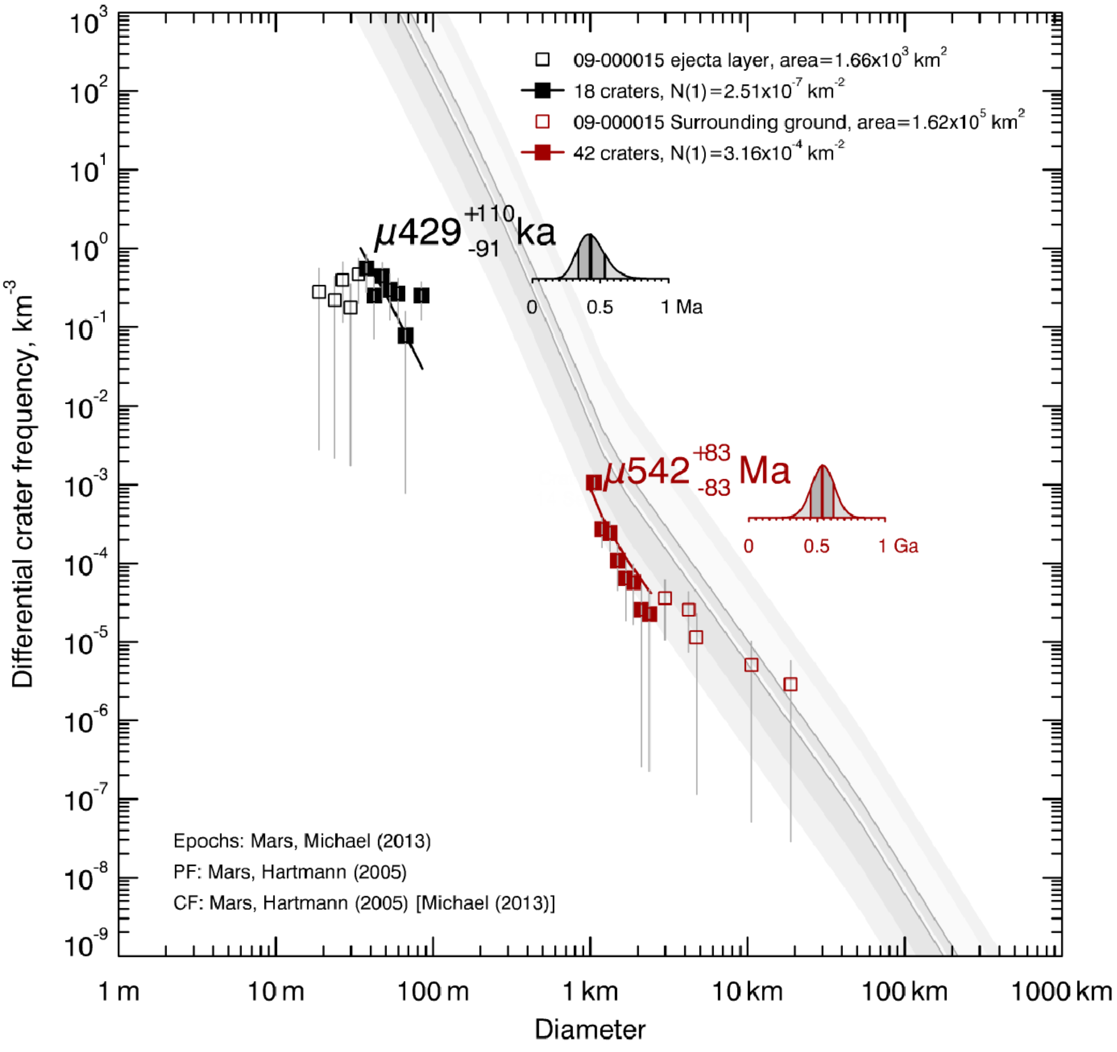

Supplementary Figure 31. Crater count and model age derivation results of 09-000015 crater. Black CSFD corresponds to craters superposed on the ejecta blanket. The red CSFD is obtained from craters superposed to the immediate vicinity of its surrounding terrain. CSFDs are displayed as differential crater frequency versus diameter[9,10]. The error bars are dependent of the number of craters counted within each diameter bin. The Poisson timing technique has been used to fit an isochrone with the observed CSFD[4]. Note that this fitting technique is independent of the binning technique used to compute the CSFD[3]. An artificial discrepancy can therefore be observed in some cases but do not result in a misinterpretation of the data[4]. Grey isochones correspond to the limit between martian epochs, from the left to the right: late Amazonian – middle Amazonian, middle Noachian – early Noachian[3,5].

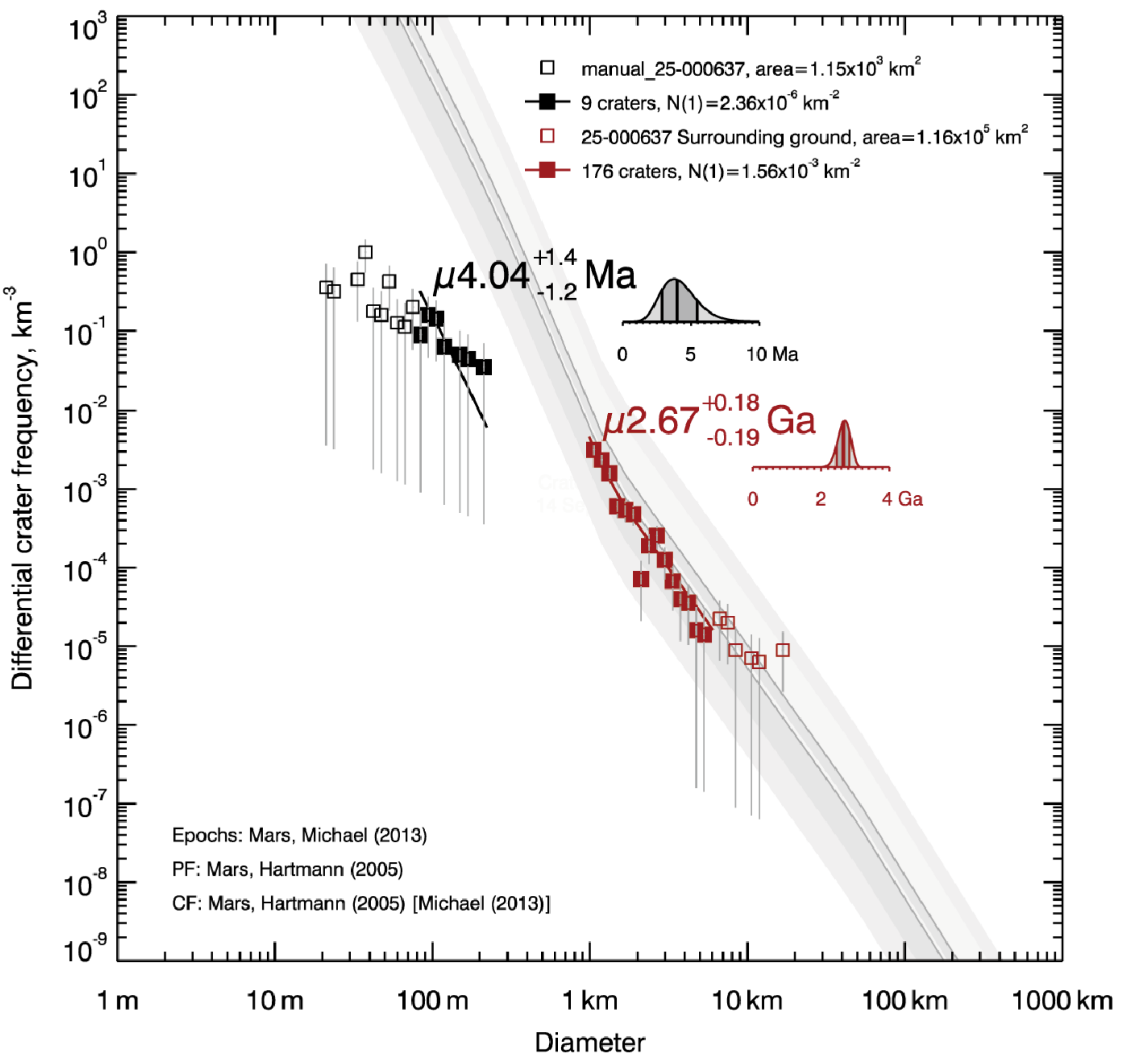

Supplementary Figure 32. Crater count and model age derivation results of 25-000637 crater. Black CSFD corresponds to craters superposed on the ejecta blanket. The red CSFD is obtained from craters superposed to the immediate vicinity of its surrounding terrain. CSFDs are displayed as differential crater frequency versus diameter[9,10]. The error bars are dependent of the number of craters counted within each diameter bin. The Poisson timing technique has been used to fit an isochrone with the observed CSFD[4]. Note that this fitting technique is independent of the binning technique used to compute the CSFD[3]. An artificial discrepancy can therefore be observed in some cases but do not result in a misinterpretation of the data[4]. Grey isochones correspond to the limit between martian epochs, from the left to the right: late Amazonian – middle Amazonian, middle Noachian – early Noachian[3,5].

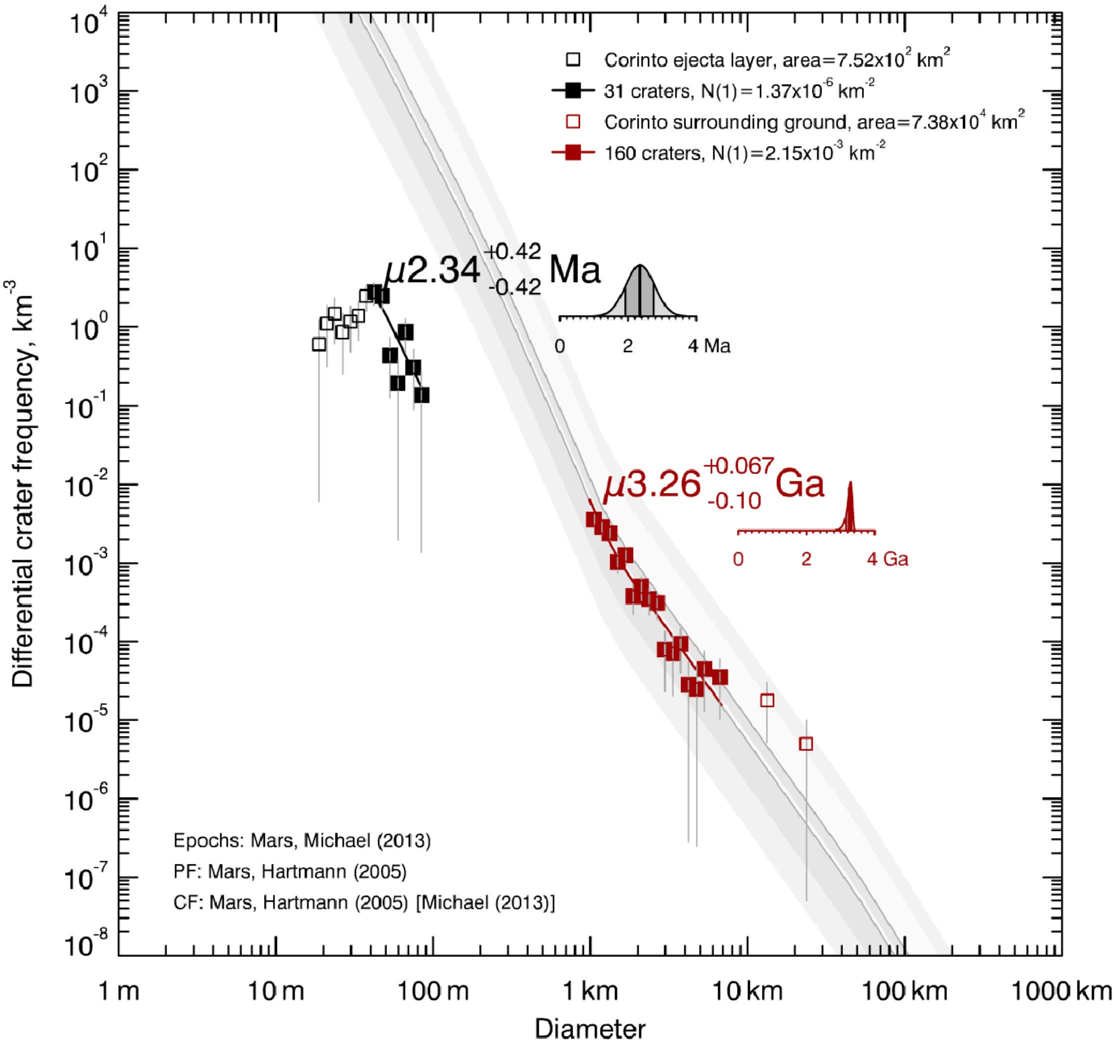

Supplementary Figure 33. Crater count and model age derivation results of Corinto crater. Black CSFD corresponds to craters superposed on the ejecta blanket. The red CSFD is obtained from craters superposed to the immediate vicinity of its surrounding terrain. CSFDs are displayed as differential crater frequency versus diameter[9,10]. The error bars are dependent of the number of craters counted within each diameter bin. The Poisson timing technique has been used to fit an isochrone with the observed CSFD[4]. Note that this fitting technique is independent of the binning technique used to compute the CSFD[3]. An artificial discrepancy can therefore be observed in some cases but do not result in a misinterpretation of the data[4]. Grey isochones correspond to the limit between martian epochs, from the left to the right: late Amazonian – middle Amazonian, middle Noachian – early Noachian[3,5].

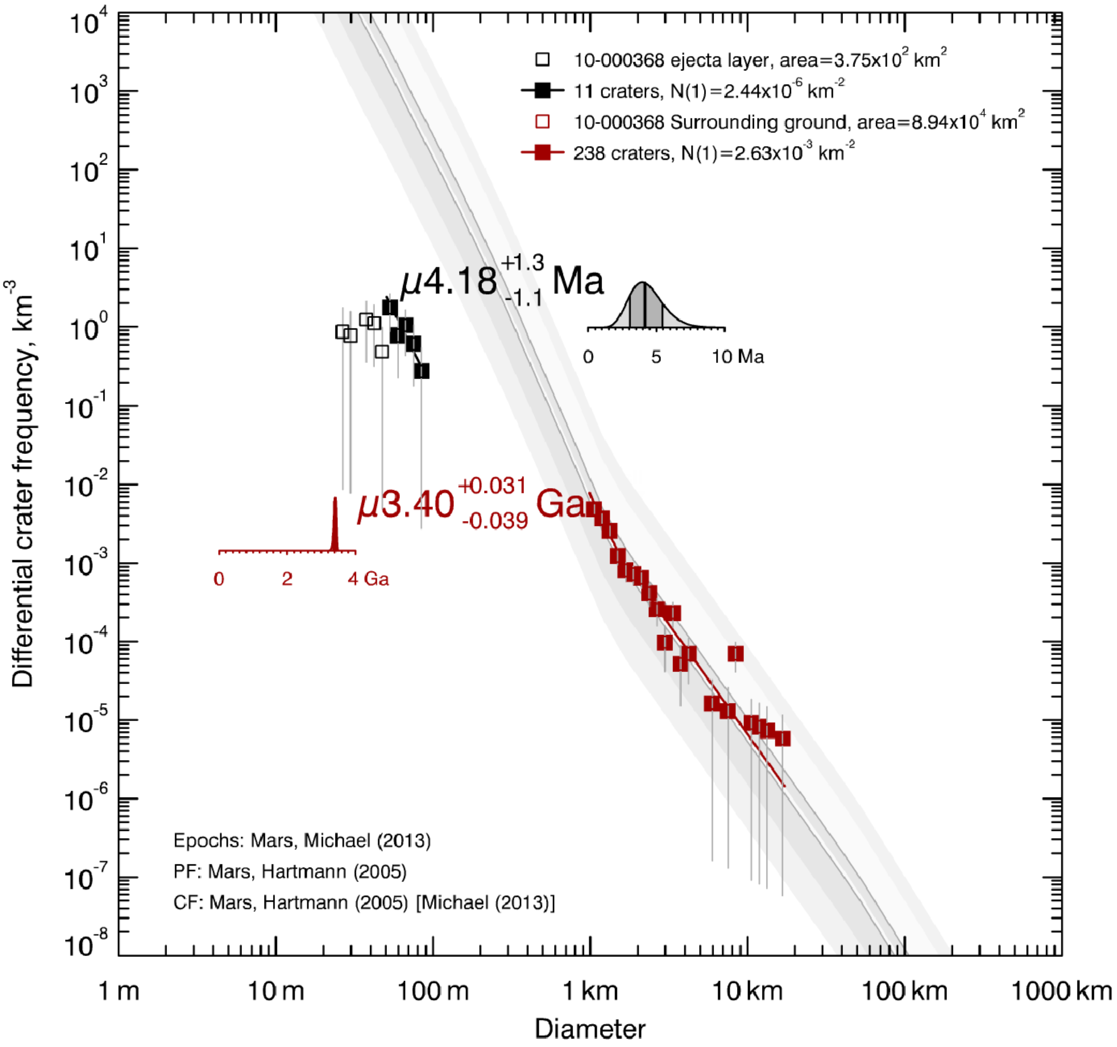

Supplementary Figure 34. Crater count and model age derivation results of Canala crater. Black CSFD corresponds to craters superposed on the ejecta blanket. The red CSFD is obtained from craters superposed to the immediate vicinity of its surrounding terrain. CSFDs are displayed as differential crater frequency versus diameter[9,10]. The error bars are dependent of the number of craters counted within each diameter bin. The Poisson timing technique has been used to fit an isochrone with the observed CSFD[4]. Note that this fitting technique is independent of the binning technique used to compute the CSFD[3]. An artificial discrepancy can therefore be observed in some cases but do not result in a misinterpretation of the data[4]. Grey isochones correspond to the limit between martian epochs, from the left to the right: late Amazonian – middle Amazonian, middle Noachian – early Noachian[3,5].

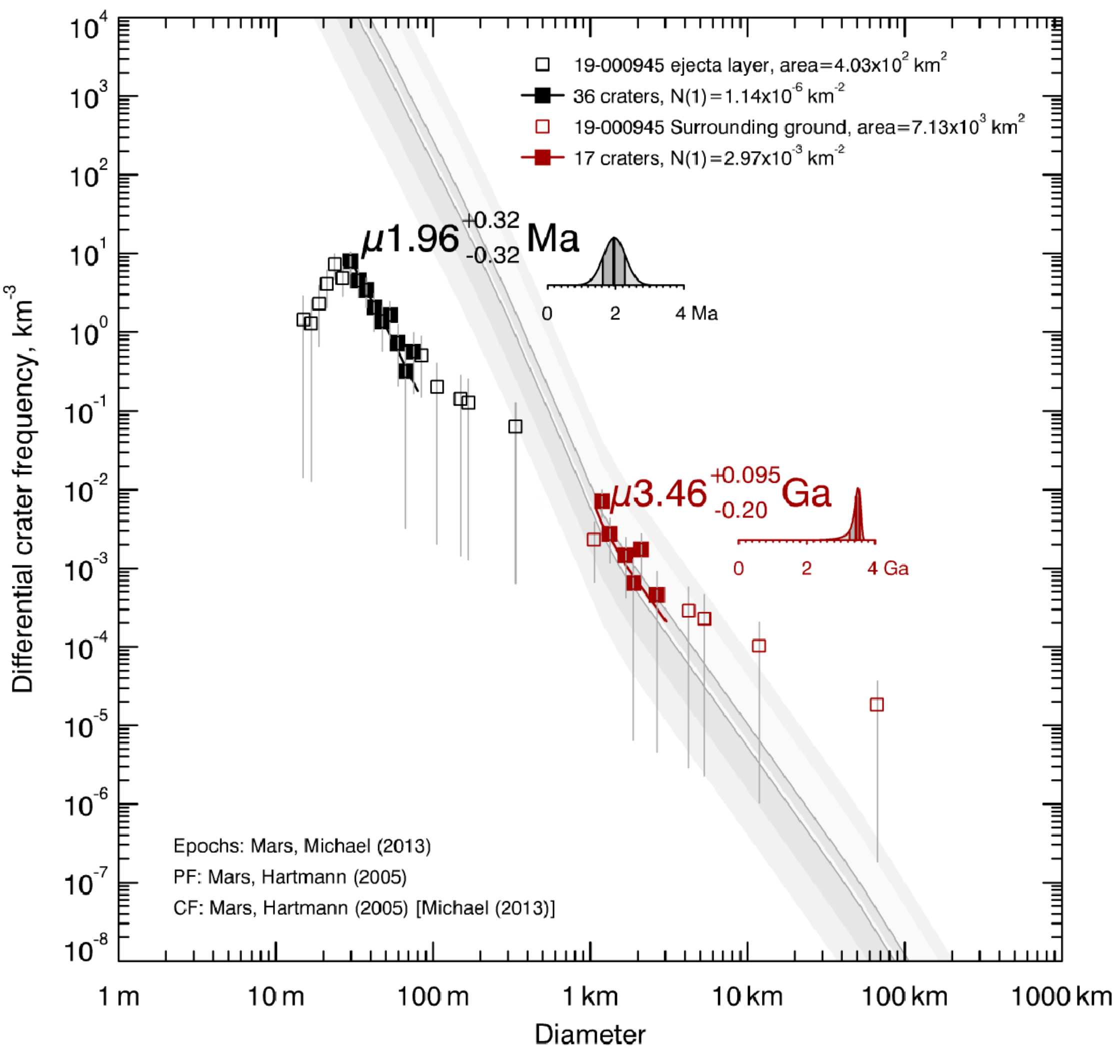

Supplementary Figure 35. Crater count and model age derivation results of 19-000945 crater. Black CSFD corresponds to craters superposed on the ejecta blanket. The red CSFD is obtained from craters superposed to the immediate vicinity of its surrounding terrain. CSFDs are displayed as differential crater frequency versus diameter[9,10]. The error bars are dependent of the number of craters counted within each diameter bin. The Poisson timing technique has been used to fit an isochrone with the observed CSFD[4]. Note that this fitting technique is independent of the binning technique used to compute the CSFD[3]. An artificial discrepancy can therefore be observed in some cases but do not result in a misinterpretation of the data[4]. Grey isochones correspond to the limit between martian epochs, from the left to the right: late Amazonian – middle Amazonian, middle Noachian – early Noachian[3,5].

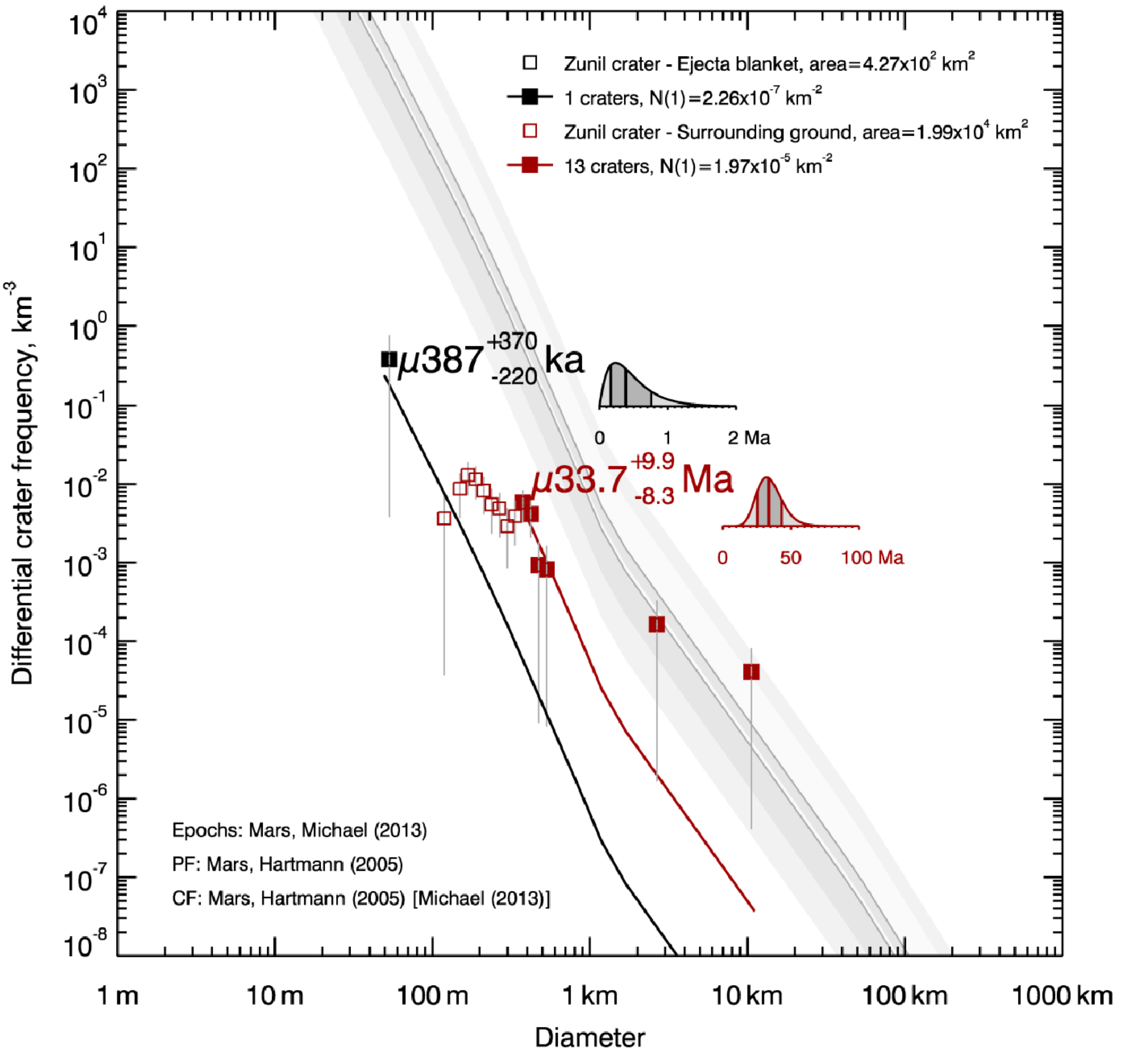

Supplementary Figure 36. Crater count and model age derivation results of Zunil crater. Black CSFD corresponds to craters superposed on the ejecta blanket. The red CSFD is obtained from craters superposed to the immediate vicinity of its surrounding terrain. CSFDs are displayed as differential crater frequency versus diameter[9,10]. The error bars are dependent of the number of craters counted within each diameter bin. The Poisson timing technique has been used to fit an isochrone with the observed CSFD[4]. Note that this fitting technique is independent of the binning technique used to compute the CSFD[3]. An artificial discrepancy can therefore be observed in some cases but do not result in a misinterpretation of the data[4]. Grey isochones correspond to the limit between martian epochs, from the left to the right: late Amazonian – middle Amazonian, middle Noachian – early Noachian[3,5].

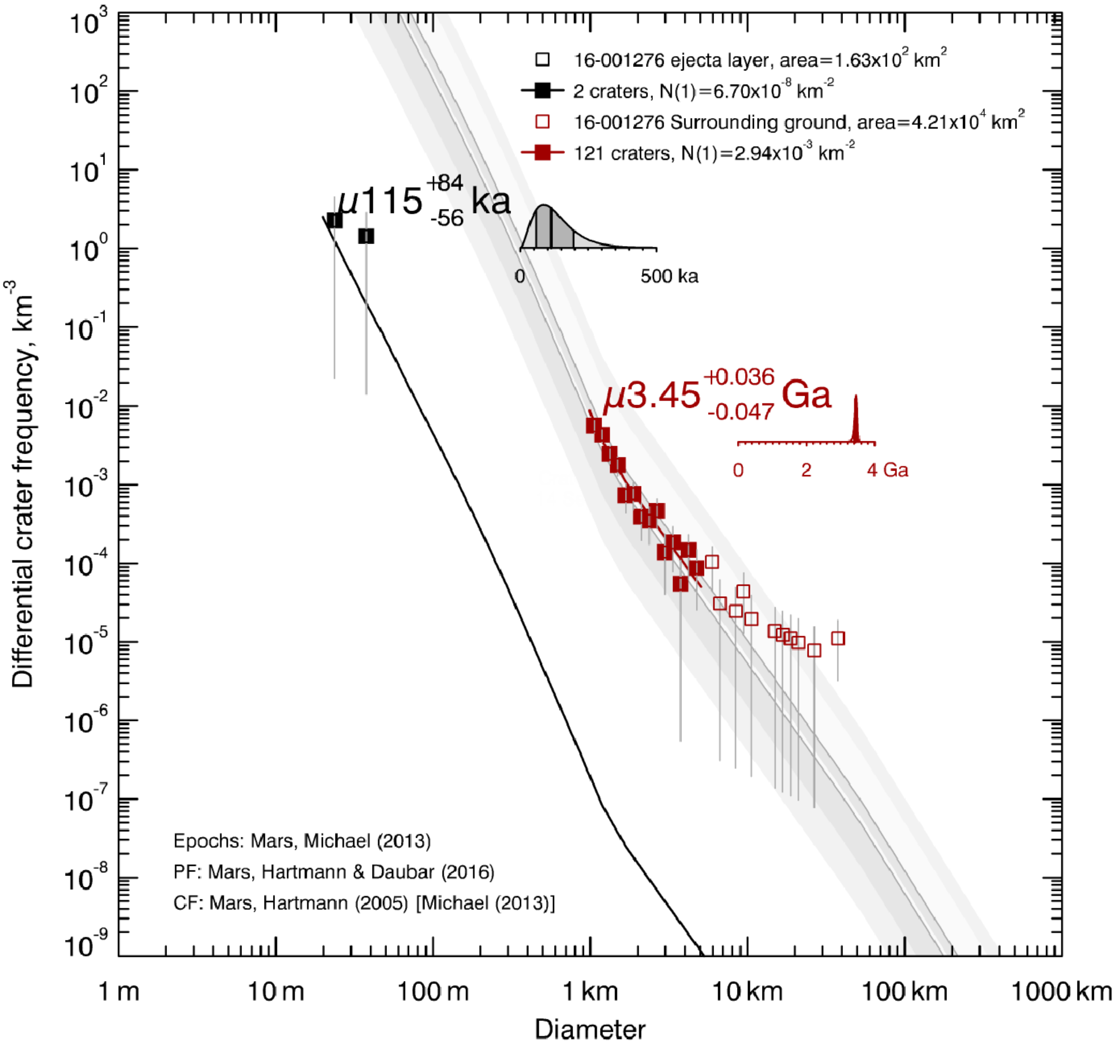

Supplementary Figure 37. Crater count and model age derivation results of 16-001276 crater. Black CSFD corresponds to craters superposed on the ejecta blanket. The red CSFD is obtained from craters superposed to the immediate vicinity of its surrounding terrain. CSFDs are displayed as differential crater frequency versus diameter[9,10]. The error bars are dependent of the number of craters counted within each diameter bin. The Poisson timing technique has been used to fit an isochrone with the observed CSFD[4]. Note that this fitting technique is independent of the binning technique used to compute the CSFD[3]. An artificial discrepancy can therefore be observed in some cases but do not result in a misinterpretation of the data[4]. Grey isochones correspond to the limit between martian epochs, from the left to the right: late Amazonian – middle Amazonian, middle Noachian – early Noachian[3,5].

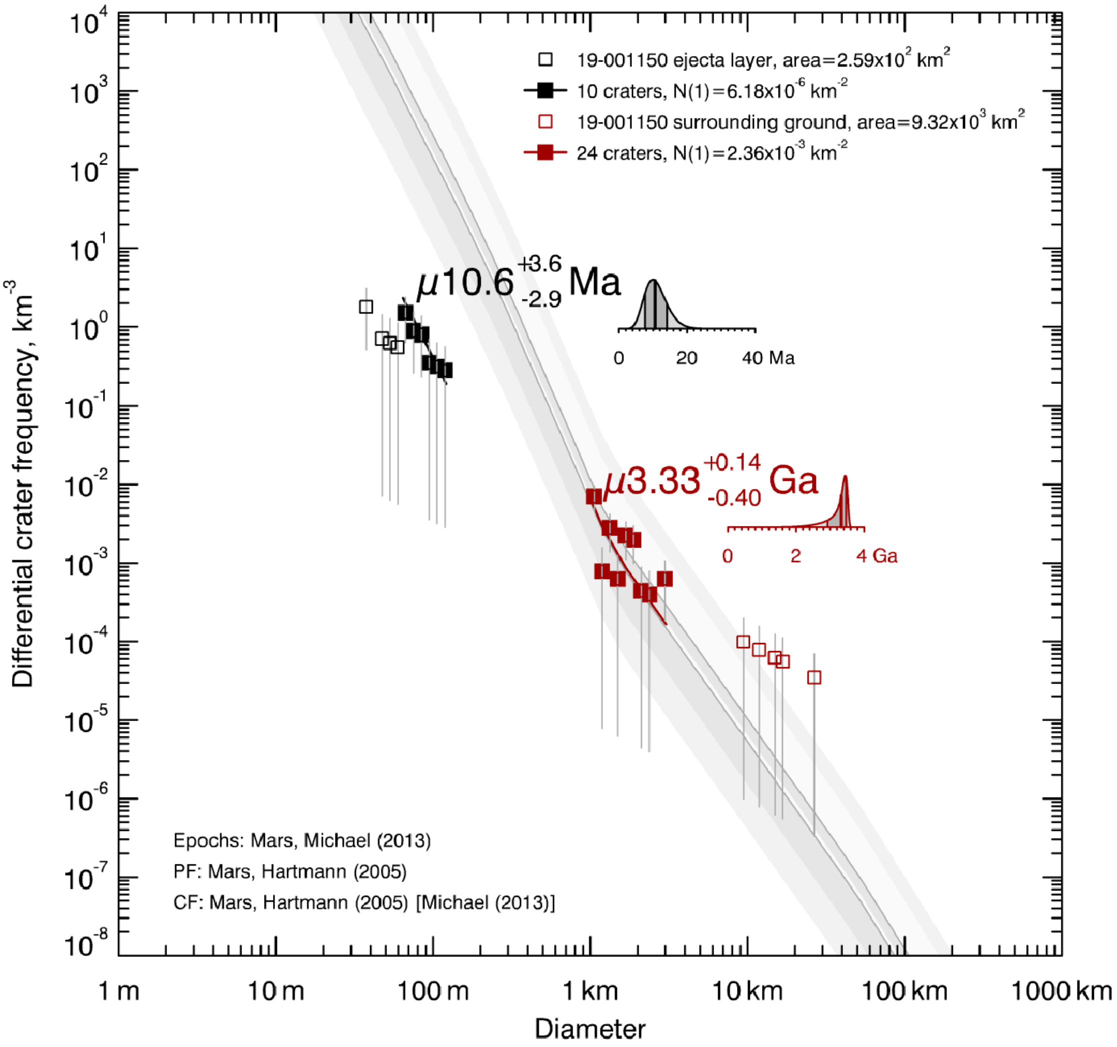

Supplementary Figure 38. Crater count and model age derivation results of 19-001150 crater. Black CSFD corresponds to craters superposed on the ejecta blanket. The red CSFD is obtained from craters superposed to the immediate vicinity of its surrounding terrain. CSFDs are displayed as differential crater frequency versus diameter[9,10]. The error bars are dependent of the number of craters counted within each diameter bin. The Poisson timing technique has been used to fit an isochrone with the observed CSFD[4]. Note that this fitting technique is independent of the binning technique used to compute the CSFD[3]. An artificial discrepancy can therefore be observed in some cases but do not result in a misinterpretation of the data[4]. Grey isochones correspond to the limit between martian epochs, from the left to the right: late Amazonian – middle Amazonian, middle Noachian – early Noachian[3,5].

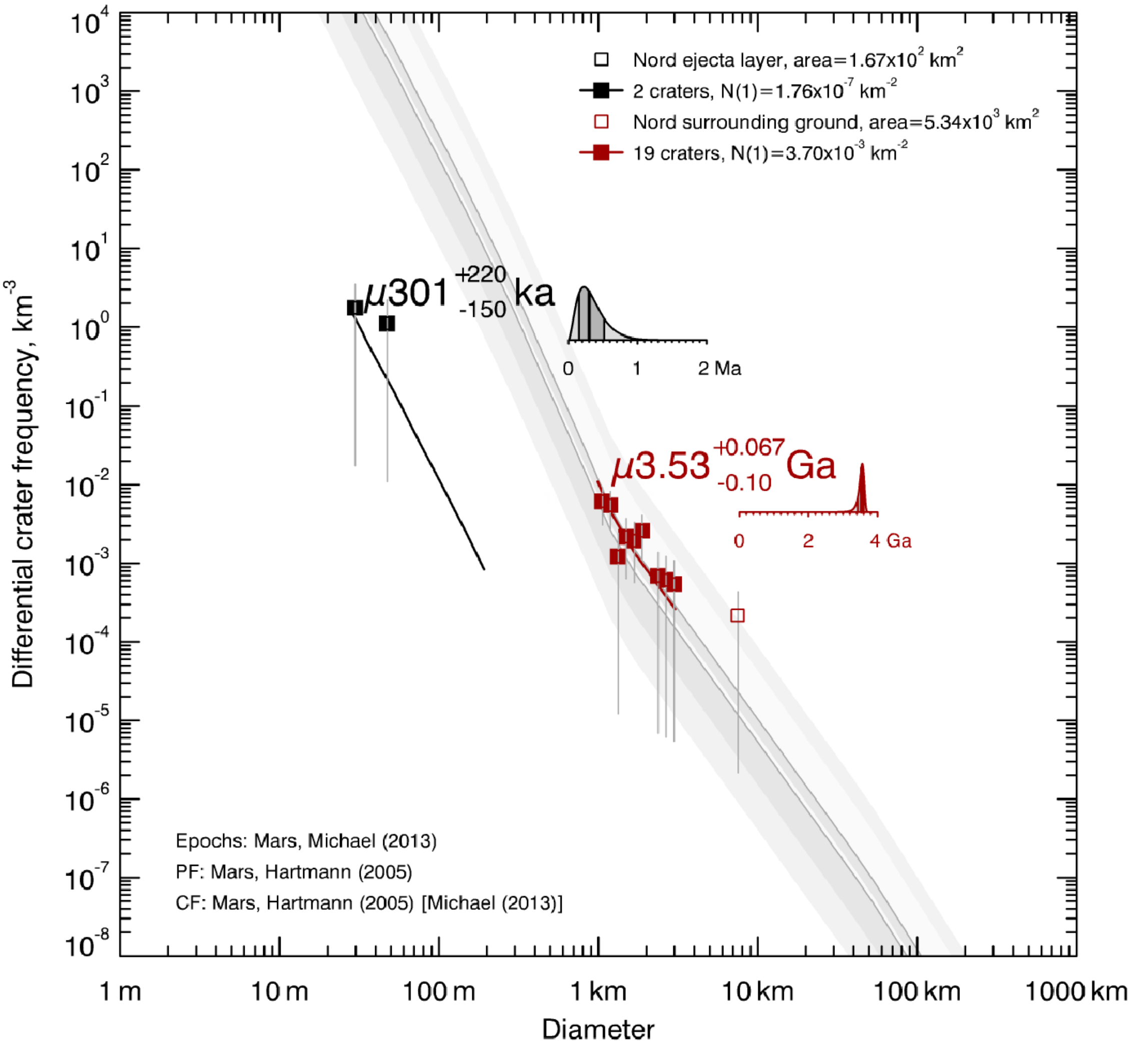

Supplementary Figure 39. Crater count and model age derivation results of Noord crater. Black CSFD corresponds to craters superposed on the ejecta blanket. The red CSFD is obtained from craters superposed to the immediate vicinity of its surrounding terrain. CSFDs are displayed as differential crater frequency versus diameter[9,10]. The error bars are dependent of the number of craters counted within each diameter bin. The Poisson timing technique has been used to fit an isochrone with the observed CSFD[4]. Note that this fitting technique is independent of the binning technique used to compute the CSFD[3]. An artificial discrepancy can therefore be observed in some cases but do not result in a misinterpretation of the data[4]. Grey isochones correspond to the limit between martian epochs, from the left to the right: late Amazonian – middle Amazonian, middle Noachian – early Noachian[3,5].

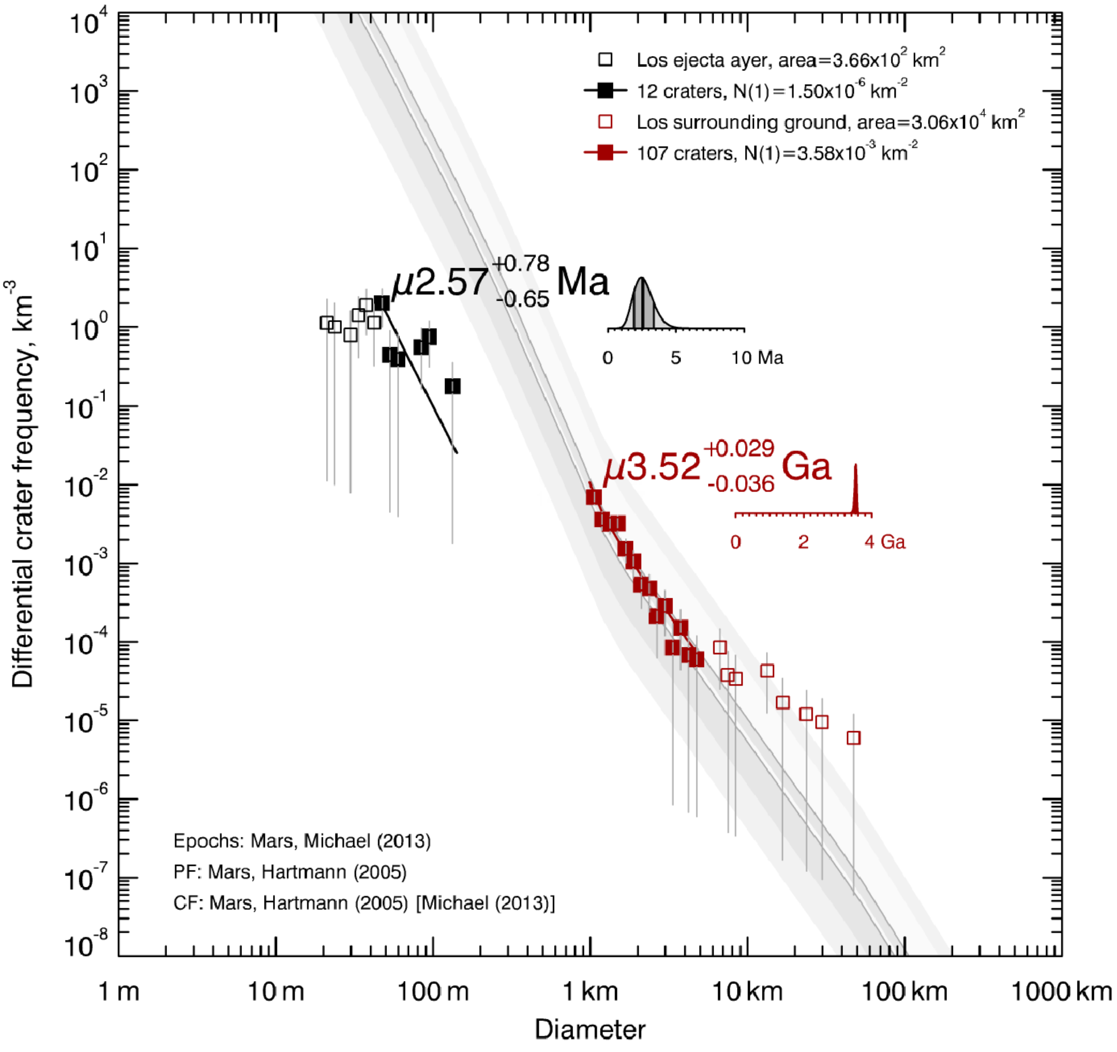

Supplementary Figure 40. Crater count and model age derivation results of Los crater. Black CSFD corresponds to craters superposed on the ejecta blanket. The red CSFD is obtained from craters superposed to the immediate vicinity of its surrounding terrain. CSFDs are displayed as differential crater frequency versus diameter[9,10]. The error bars are dependent of the number of craters counted within each diameter bin. The Poisson timing technique has been used to fit an isochrone with the observed CSFD[4]. Note that this fitting technique is independent of the binning technique used to compute the CSFD[3]. An artificial discrepancy can therefore be observed in some cases but do not result in a misinterpretation of the data[4]. Grey isochones correspond to the limit between martian epochs, from the left to the right: late Amazonian – middle Amazonian, middle Noachian – early Noachian[3,5].

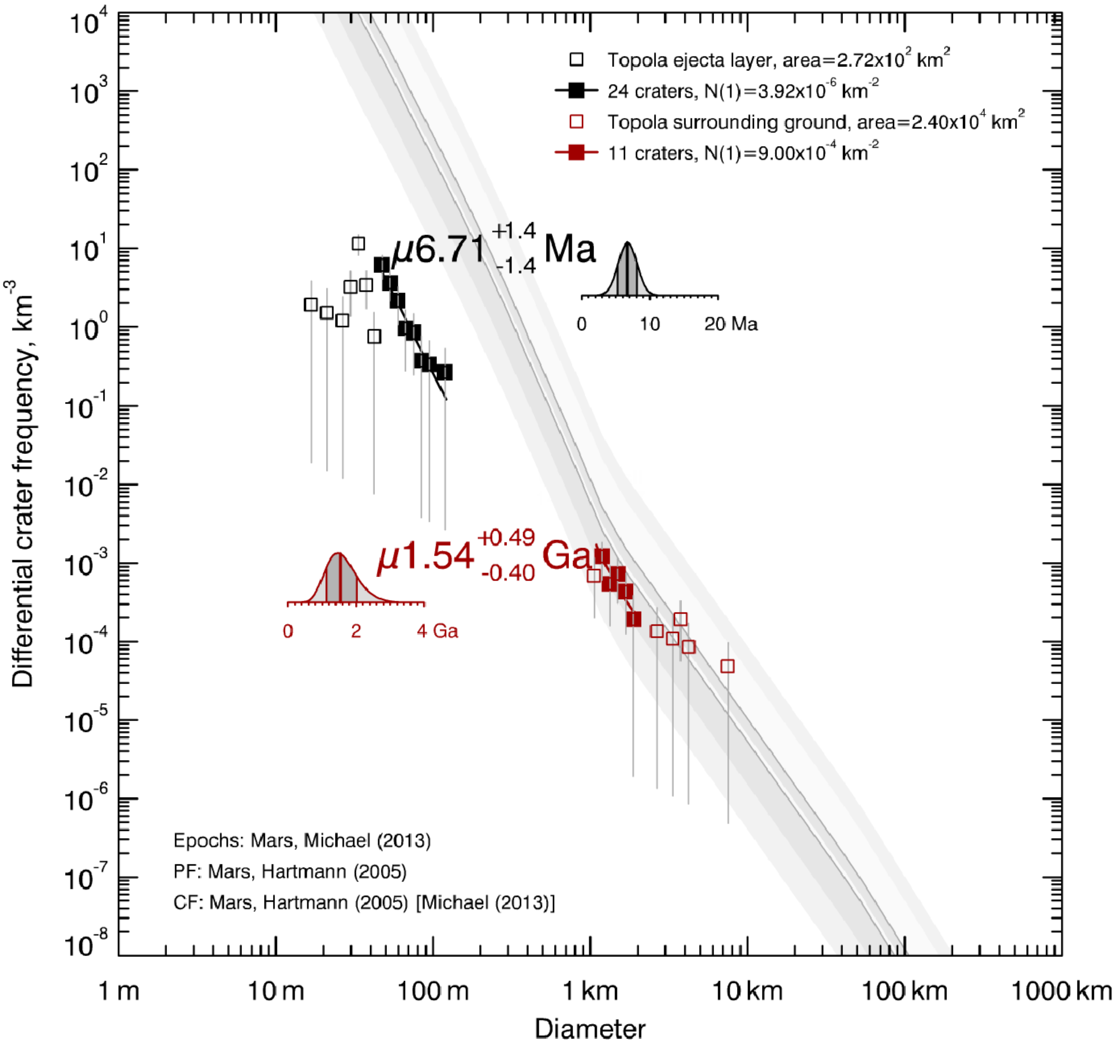

Supplementary Figure 41. Crater count and model age derivation results of Topola crater. Black CSFD corresponds to craters superposed on the ejecta blanket. The red CSFD is obtained from craters superposed to the immediate vicinity of its surrounding terrain. CSFDs are displayed as differential crater frequency versus diameter[9,10]. The error bars are dependent of the number of craters counted within each diameter bin. The Poisson timing technique has been used to fit an isochrone with the observed CSFD[4]. Note that this fitting technique is independent of the binning technique used to compute the CSFD[3]. An artificial discrepancy can therefore be observed in some cases but do not result in a misinterpretation of the data[4]. Grey isochones correspond to the limit between martian epochs, from the left to the right: late Amazonian – middle Amazonian, middle Noachian – early Noachian[3,5].

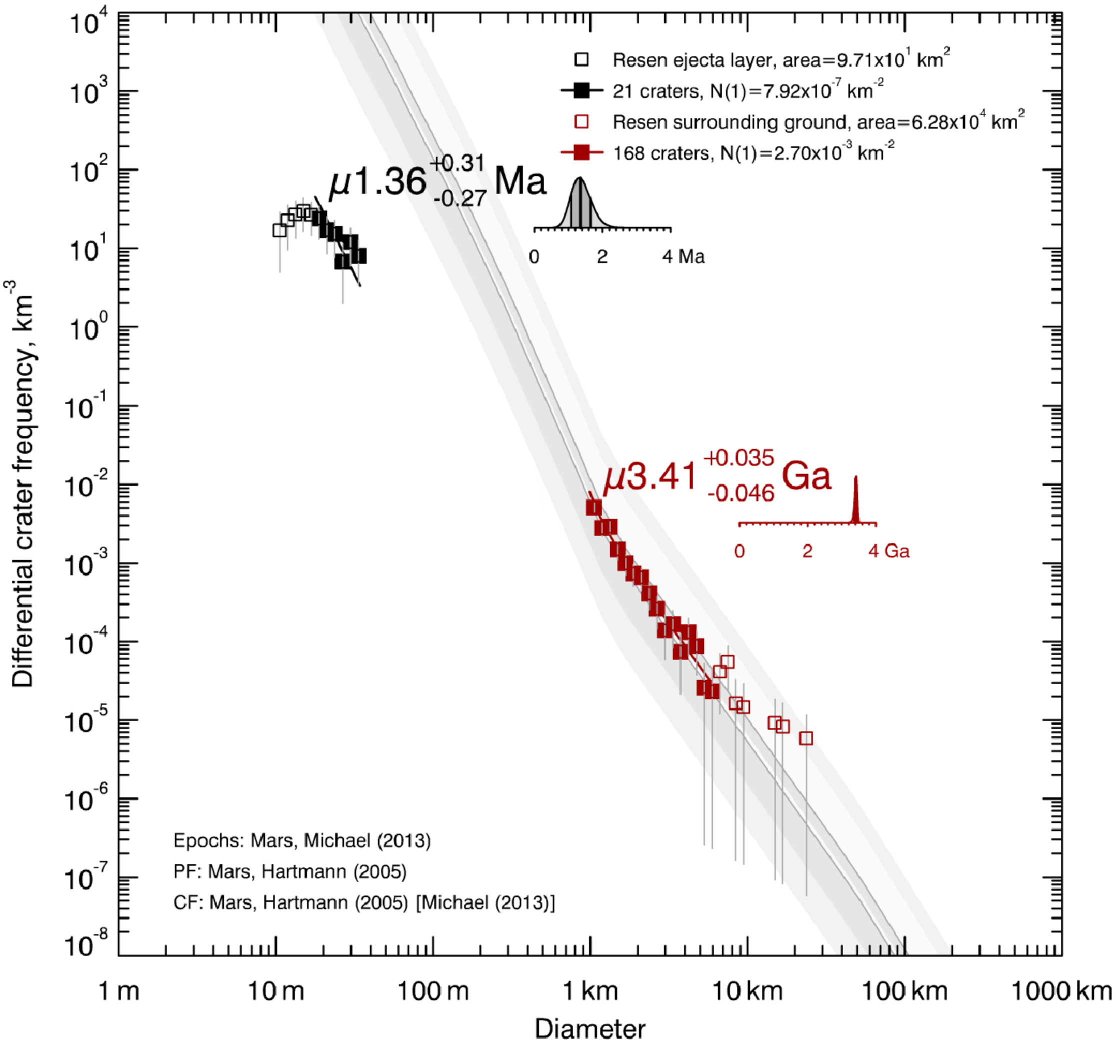

Supplementary Figure 42. Crater count and model age derivation results of Resen crater. Black CSFD corresponds to craters superposed on the ejecta blanket. The red CSFD is obtained from craters superposed to the immediate vicinity of its surrounding terrain. CSFDs are displayed as differential crater frequency versus diameter[9,10]. The error bars are dependent of the number of craters counted within each diameter bin. The Poisson timing technique has been used to fit an isochrone with the observed CSFD[4]. Note that this fitting technique is independent of the binning technique used to compute the CSFD[3]. An artificial discrepancy can therefore be observed in some cases but do not result in a misinterpretation of the data[4]. Grey isochones correspond to the limit between martian epochs, from the left to the right: late Amazonian – middle Amazonian, middle Noachian – early Noachian[3,5].

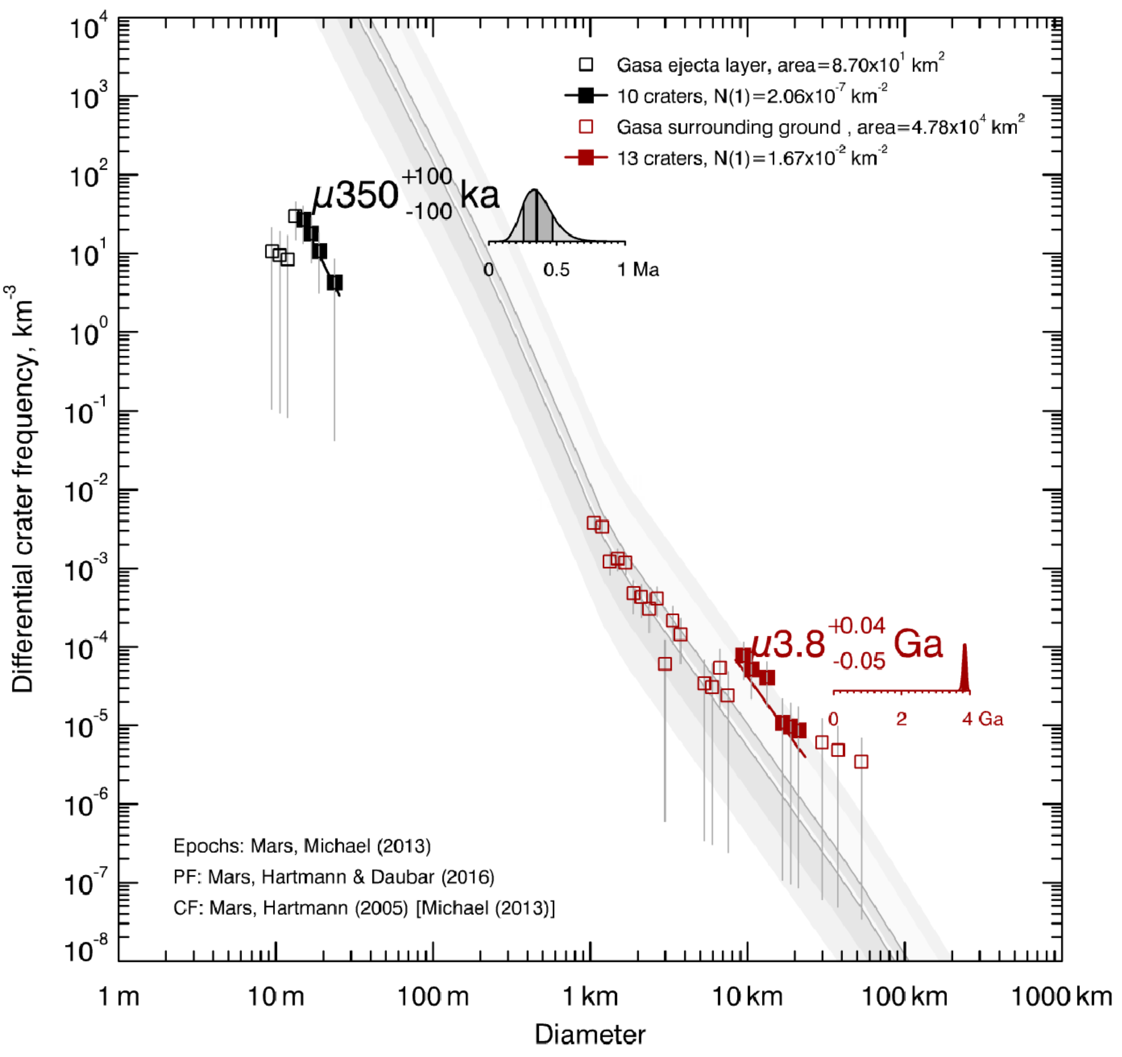

Supplementary Figure 43. Crater count and model age derivation results of Gasa crater. Black CSFD corresponds to craters superposed on the ejecta blanket. The red CSFD is obtained from craters superposed to the immediate vicinity of its surrounding terrain. CSFDs are displayed as differential crater frequency versus diameter[9,10]. The error bars are dependent of the number of craters counted within each diameter bin. The Poisson timing technique has been used to fit an isochrone with the observed CSFD[4]. Note that this fitting technique is independent of the binning technique used to compute the CSFD[3]. An artificial discrepancy can therefore be observed in some cases but do not result in a misinterpretation of the data[4]. Grey isochones correspond to the limit between martian epochs, from the left to the right: late Amazonian – middle Amazonian, middle Noachian – early Noachian[3,5].

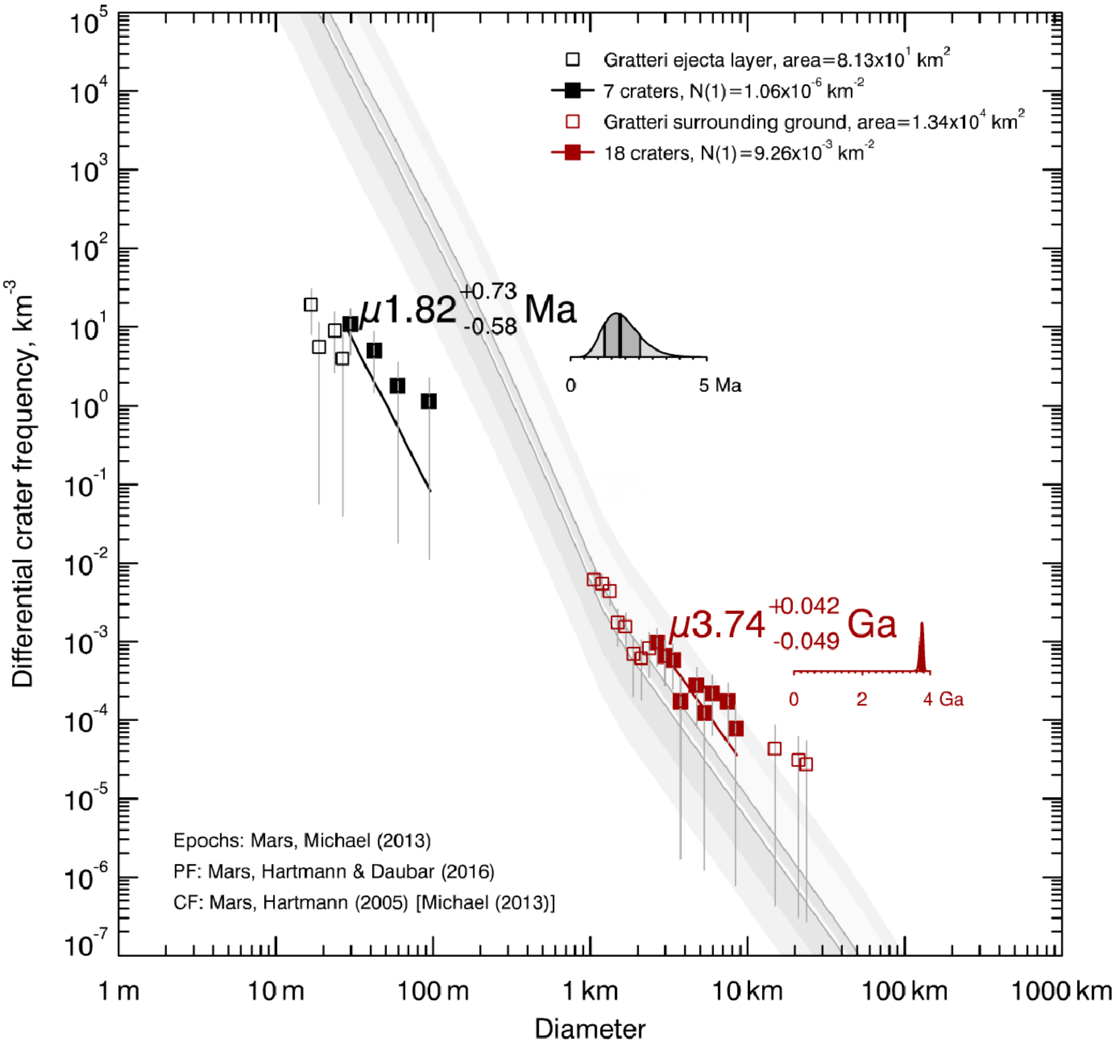

Supplementary Figure 44. Crater count and model age derivation results of Gratteri crater. Black CSFD corresponds to craters superposed on the ejecta blanket. The red CSFD is obtained from craters superposed to the immediate vicinity of its surrounding terrain. CSFDs are displayed as differential crater frequency versus diameter[9,10]. The error bars are dependent of the number of craters counted within each diameter bin. The Poisson timing technique has been used to fit an isochrone with the observed CSFD[4]. Note that this fitting technique is independent of the binning technique used to compute the CSFD[3]. An artificial discrepancy can therefore be observed in some cases but do not result in a misinterpretation of the data[4]. Grey isochones correspond to the limit between martian epochs, from the left to the right: late Amazonian – middle Amazonian, middle Noachian – early Noachian[3,5].

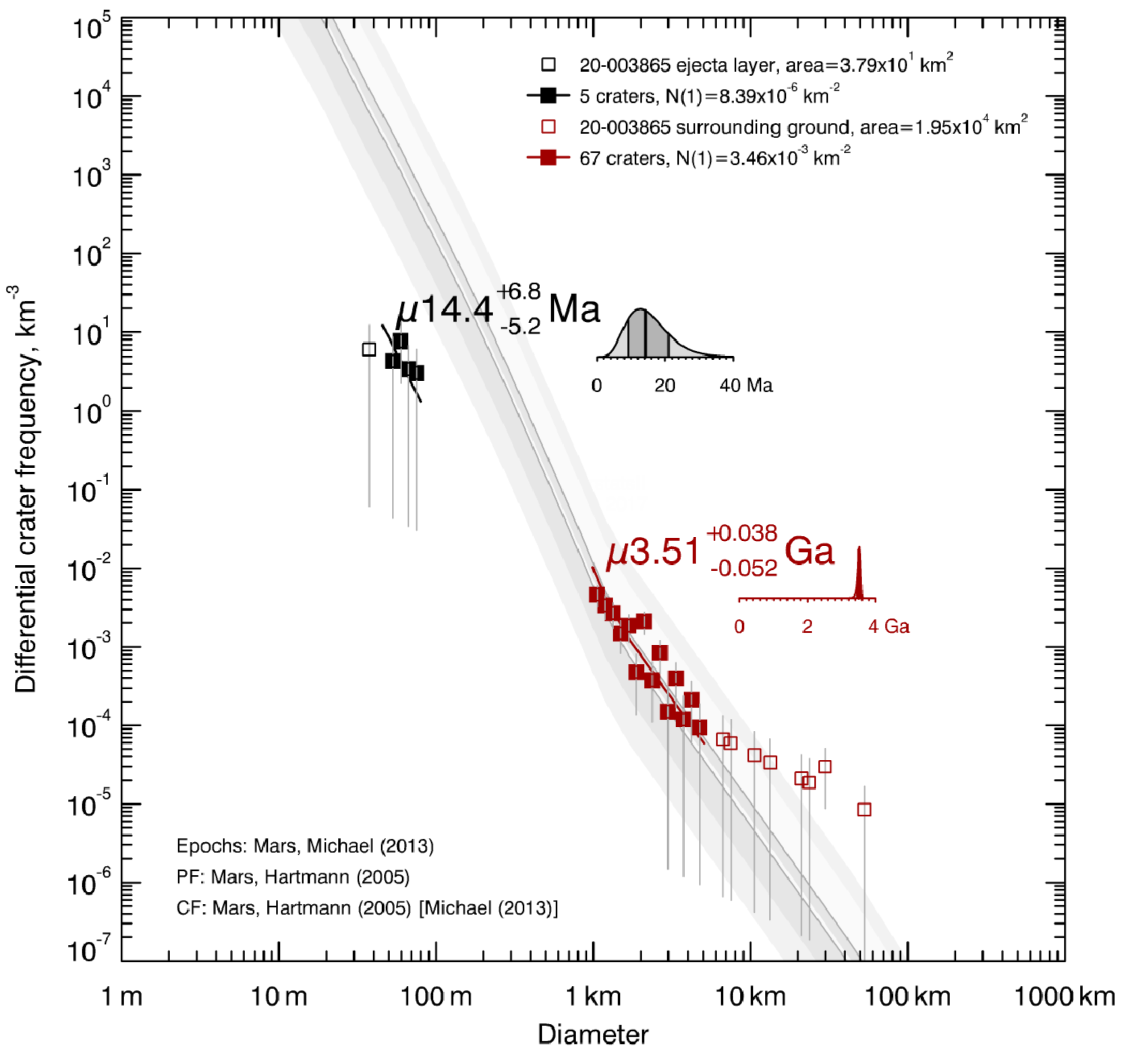

Supplementary Figure 45. Crater count and model age derivation results of 20-003865 crater. Black CSFD corresponds to craters superposed on the ejecta blanket. The red CSFD is obtained from craters superposed to the immediate vicinity of its surrounding terrain. CSFDs are displayed as differential crater frequency versus diameter[9,10]. The error bars are dependent of the number of craters counted within each diameter bin. The Poisson timing technique has been used to fit an isochrone with the observed CSFD[4]. Note that this fitting technique is independent of the binning technique used to compute the CSFD[3]. An artificial discrepancy can therefore be observed in some cases but do not result in a misinterpretation of the data[4]. Grey isochones correspond to the limit between martian epochs, from the left to the right: late Amazonian – middle Amazonian, middle Noachian – early Noachian[3,5].

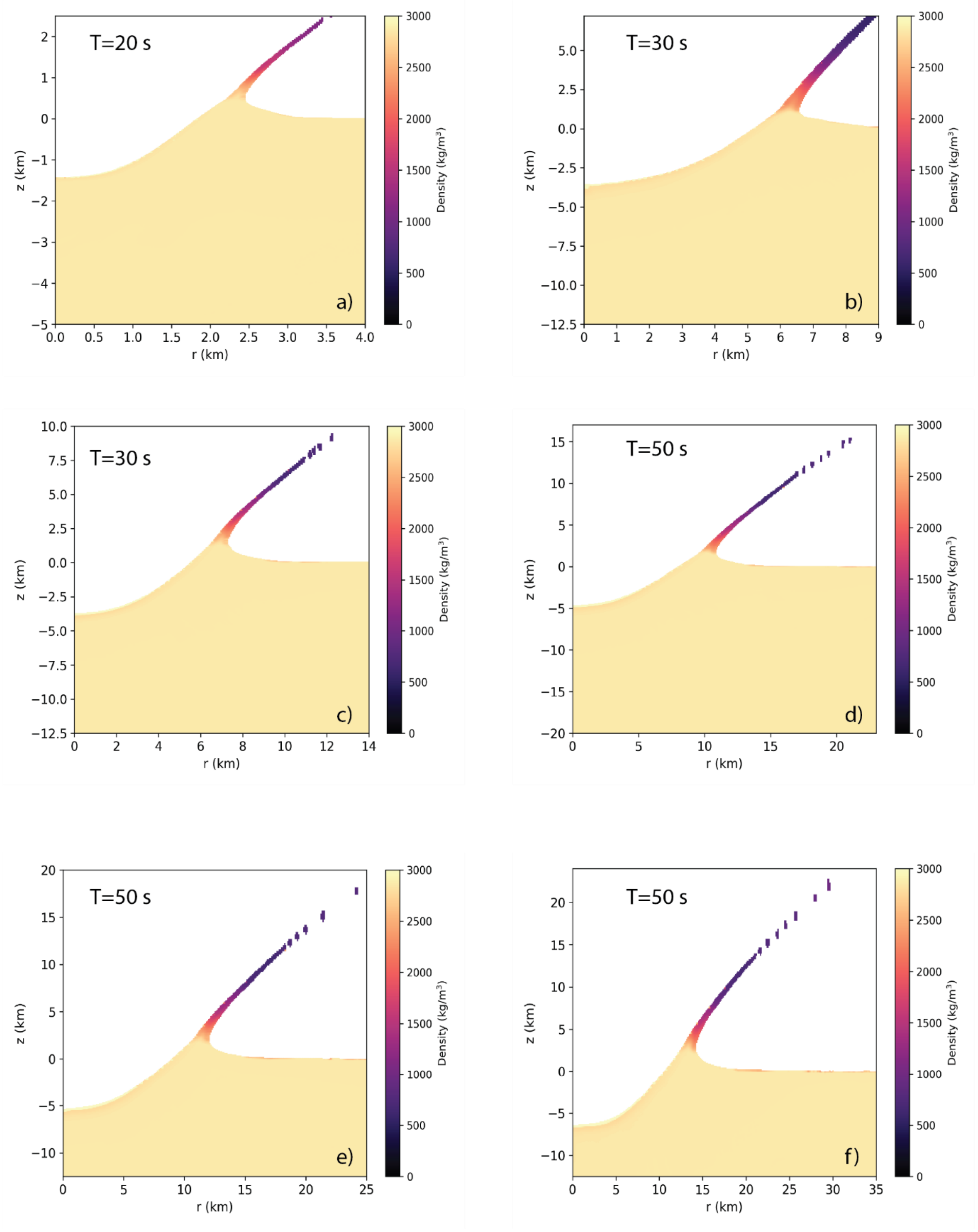

Supplementary Fig. 46. iSALE-2D simulations outputs. The moment of the transient crater (D_t) is shown for final crater diameter (D_f): a) D_f = 4.22 km (D_t=3.5 km), b) D_f = 12.24 km (D_t=8.97 km), c) D_f = 13.28 km (D_t=9.6 km), d) D_f = 20.55 km (D_t=14.19 km), e) D_f = 23.3 km (D_t=18.08 km), f) D_f = 27.98 km (D_t=18.65 km). The excavated volume for each is used for approximated scaling in Supplementary Fig. 10.

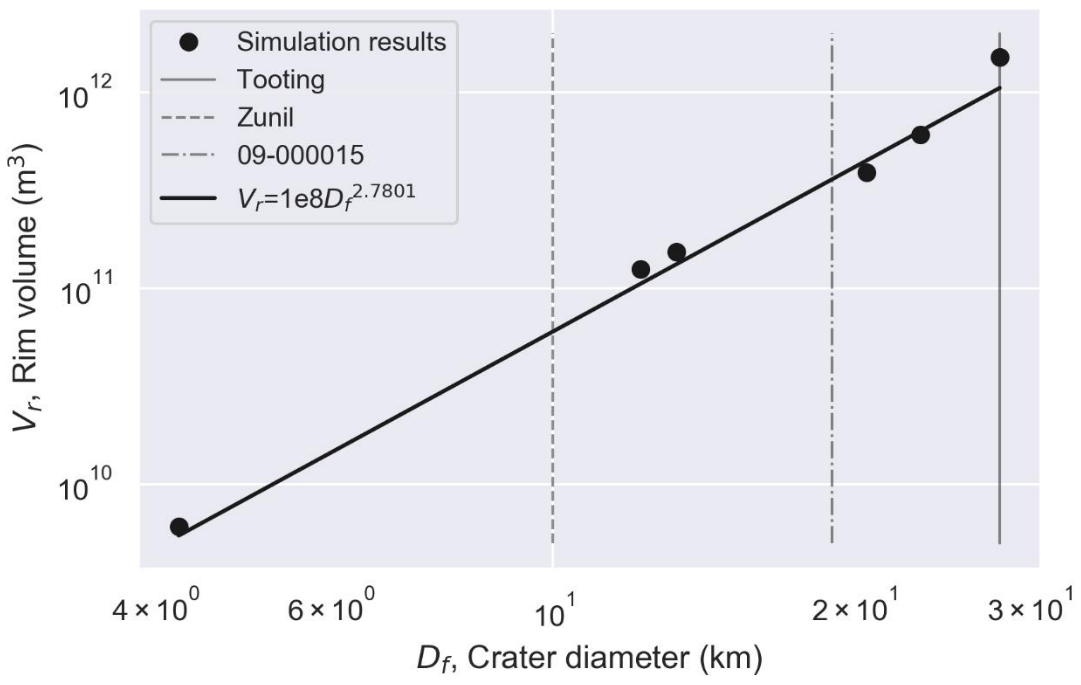

Supplementary Figure 47. Results from modelled crater showing power-law increase in transient crater rim volume with increase in the crater diameter. The transient crater rim volume is considered to be an approximation for the excavated volume. Simulation results presented in Supplementary Figure 46 are shown as black dots. Crater diameter corresponding to Tooting, 09-000015 and Zunil craters are also mentioned and symbolised as grey dashed and plain lines.

Terrain type			VOLCANIC	HIGHLANDS	LOWLANDS	APRON	IMPACT	HIGH LATITUDES	SUM
Image name			E-132_N08	E024_N-20	E-036_N28 & E-032_N28	E-144_N24	E092_N08	E116_N48	
Manual counting	GT	Total	437	270	579	274	141	223	1924
		> 60 m	73	175	309	123	113	131	924
		> 70 m	39	152	232	77	94	112	706
		>100 m	11	98	120	22	50	70	371
		> 200 m	1	26	22	0	5	53	107
Crater Detection Algorithm	TP	Total	103	134	352	125	79	120	913
		> 60 m	65	117	239	90	74	92	677
		> 70 m	39	101	187	61	63	79	530
		> 100 m	11	66	101	19	42	44	283
		> 200 m	1	20	19	0	5	32	77
	FP	Total	0	3	21	2	1	32	59
		FP>60m	0	3	7	2	1	26	39
		FP>70m	0	3	5	1	1	22	32
		FP>100m	0	2	1	0	0	8	11
		FP>200m	0	0	0	0	0	4	4
	FN	Total	334	136	227	149	62	103	1011
		> 60 m	8	58	70	33	39	39	247
		> 70 m	0	51	45	16	31	33	176
		> 100 m	0	32	19	3	8	26	88
		> 200 m	0	6	3	0	0	21	30
Metrics	Recall	Total	0.24	0.50	0.61	0.46	0.56	0.54	0.47
		> 60 m	0.89	0.67	0.77	0.73	0.65	0.70	0.73
		> 70 m	1.00	0.66	0.81	0.79	0.67	0.71	0.75
		> 100 m	1.00	0.67	0.84	0.86	0.84	0.63	0.76
		> 200 m	1.00	0.77	0.86	/	1.00	0.60	0.72
	Precision	Total	1.00	0.98	0.94	0.98	0.99	0.79	0.94
		> 60 m	1.00	0.98	0.97	0.98	0.99	0.78	0.95
		> 70 m	1.00	0.97	0.97	0.98	0.98	0.78	0.94
		> 100 m	1.00	0.97	0.99	1.00	1.00	0.85	0.96
		> 200 m	1.00	1.00	1.00	/	1.00	0.89	0.95
	F1 score	Total	0.38	0.66	0.74	0.62	0.71	0.64	0.63
		> 60 m	0.94	0.79	0.86	0.84	0.79	0.74	0.83
		> 70 m	1.00	0.79	0.88	0.88	0.80	0.74	0.84
		> 100 m	1.00	0.80	0.91	0.93	0.91	0.72	0.85
		> 200 m	1.00	0.87	0.93	/	1.00	0.72	0.82

Supplementary Table 1. Confusion matrix of CDA's detections over each evaluation test area and splitted by diameter. The name of the image where the evaluation is performed is indicated for each area[11] (see Supplementary Figure 1 for their location). Craters that are manually counted (considered here as Ground Truth (GT)) but are not found by the algorithm are False Negative (FN) and craters which the algorithm locates but are not manually identified are False Positive (FP). True Positive (TP) are craters that are both manually identified and detected by the algorithm. For a definition of he three metrics presented here (recall, precision, and F1 score) the readers are referred to the Application to the CTX global mosaic and evaluation subsection of the Methods.

Crater						Surrounding terrain		
Name[2] / Label	Latitude	Longitude	Diameter (km)	Model Age (Ma)		Unit[8]	Model age (Ga)	
Mojave (1)	7.5	-33.0	58	1.7	+0.2 -0.2	mNh, Ht	3.57 (mNh)	+0.038 -0.05
Kotka (2)	19.3	169.9	39	22.8	+7.7 -6.8	HNt	2.21	0.22 0.22
Tooting (3)	23.2	-152.2	29	0.957	+0.26 -0.22	lAv	0.308	+0.041 -0.041
12-000681 (4)	5.2	21.2	19	2.63	+0.37 -0.37	mNh	3.03	+0.16 -0.24
09-000015 (5)	19.3	-99.9	19	0.429	+0.11 -0.091	AHv	0.542	+0.083 -0.083
25-000637 (6)	-43.5	-101.5	17	4.04	+1.4 -1.2	eHv	2.67	+0.18 -0.19
Corinto (7)	16.9	141.7	14	2.34	+0.42 -0.42	AHv	3.26	+0.067 -0.1
Canala (8)	24.4	-80.1	11	4.18	+1.3 -1.1	eHh	3.4	+0.031 -0.039
19-000945 (9)	-19.7	-3.3	11	1.96	+0.32 -0.32	eNh	3.46	+0.095 -0.2
Zunil (10)	7.7	166.2	10	0.387	+0.37 -0.22	lAvf	0.033	+0.010 -0.008
16-001276 (11)	-15.7	-156.4	10	0.115	+0.094 -0.056	mNh	3.45	+0.036 -0.047
19-001150 (12)	-29.4	-8.7	9	10.6	+3.6 -2.9	eNh	3.33	+0.14 -0.4
Noord (13)	-19.3	-11.3	8	0.301	+0.22 -0.15	mNh	3.53	+0.067 -0.1
Los (14)	-35.1	-76.2	8	2.57	+0.78 -0.65	mNh	3.52	+0.029 -0.036
Topola (15)	15.8	-92.2	8	6.71	+1.4 -1.4	AHv	1.54	+0.49 -0.4
Resen (16)	-27.9	108.9	7	1.36	+0.31 -0.27	eHv	3.41	+0.035 -0.046
Gasa (17)	-35.7	129.4	7	0.35	+0.1 -0.1	eNh	3.8	+0.04 -0.05
Gratteri (18)	-17.7	-160.1	7	1.82	+0.73 -0.58	mNh	3.74	+0.042 -0.049
20-003865 (19)	-11.0	25.9	3	14.4	+6.8 -5.2	mNh	3.51	+0.038 -0.052

Supplementary Table 2. Location, size, and model age of crater candidates for the ejection of martian meteorites. The model age of the surrounding ground and the name of the geological unit[8] is also mentioned. Number under each crater name corresponds to the labels as shown on Figure 1.a of the main text.

Resolution (CPPR)	20	20	20	20	20	20
Grid spacing	13	35	50	75	88	113
Mesh size (number of cells in high resolution)	400X450	400X450	400X450	400X450	400X450	400X450
Mesh size (number of cells in extension zone)	50X50	50X50	50X50	50X50	50X50	50X50
End time (s)	600	600	600	600	600	600
Save interval	5	5	5	5	5	5
Impactor diameter (km)	0.5	1.4	2	3	3.5	4.5
Vertical impact velocity component at the ground (kms^{-1})	3.89	3.89	3.89	3.89	3.89	3.89
Impact angle (°)	90	90	90	90	90	90
Crater diameter (final estimate) (km)	~5	~12	~13	~21	~23	~28

Supplementary Table 3. Impact condition parameters used in iSALE simulations. The input parameters presented here have been used to produce the results presented on Supplementary Figures 46 and 47.

	Target	Projectile
Material name	Basalt	Dunite
Equation of state	ANEOS	ANEOS
Strength model	ROCK	ROCK
Damage model	IVANOV	IVANOV
Poission’s coefficient	0.3	0.25
Cohesion (damaged) (KPa)	10	10
Friction (damaged)	0.6	0.6
Limiting strength (damaged) (GPa)	3.5	3.5
Cohesion (intact) (MPa)	10	10
Friction (intact)	1.2	1.2
Limiting strength (intact) (GPa)	3.5	3.5

Supplementary Table 4. Material model parameters used to represent projectile and target in iSALE numerical models. The input parameters presented here have been used to produce the results presented on Supplementary Figures 46 and 47.

Supplementary References

1. Lagain, A. et al. Mars Crater Database: A participative project for the classification of the morphological characteristics of large Martian craters, in Reimold, W.U., and Koeberl, C., eds., *Large Meteorite Impacts and Planetary Evolution VI* **550**, p. 1-16 (2021).
2. Robbins, S. J. & Hynek, B. M. A new global database of Mars impact craters ≥1 km: 1. Database creation, properties, and parameters. *J. Geophys. Res.* **117**, E05004, (2012).
3. Michael, G. G. & Neukum, G. Planetary surface dating from crater size frequency distribution measurements: partial resurfacing events and statistical age uncertainty. *Earth Planet. Sci. Lett.* **294**, 223-229 (2010).
4. Michael, G. G., Kneissl, T. & Neesemann, A. Planetary surface dating from crater size-frequency distribution measurements: Poisson timing analysis. *Icarus* **277**, 279–285 (2016).
5. Hartmann, W. K. Martian cratering 8: Isochron refinement and the chronology of Mars. *Icarus* **174**, 294-320 (2005).
6. Lapen, T. J. et al. Two billion years of magmatism recorded from a single Mars meteorite ejection site. *Sci. Adv.* **3**(2), e1600922 (2017).
7. Udry, A. et al.. What Martian meteorites reveal about the interior and surface of Mars. *J. Geophys. Res.* **25**, e2020JE006523 (2020).
8. Tanaka, K. L., et al. Geologic map of Mars. *U.S. Geological Survey Scientific Investigations Map 3292* (2014).
9. Fassett, C. I. Analysis of impact crater populations and the geochronology of planetary surfaces in the inner solar system, *J. Geophys. Res.* **121**, 1900-1926 (2016).
10. Crater Analysis Techniques Working Group. Standard techniques for presentation and analysis of crater size-frequency data. *Icarus*, **37**, 467–474 (1979).
11. Dickson, J. L., Kerber, L. A., Fassett, C.I. & Ehlmann, B. L. A global, blended CTX mosaic of Mars with vectorized seam mapping. A new mosaicking pipeline using principles of non-destructive image editing. *49th Lunar and Plan. Sci. Conf.* (2019).
